# Storage-Dependent Responses of KDML 105 Rice (*Oryza sativa* L.) Seeds Across Dynamic Magnetic-Field Treatment Conditions: Germination, Seedling Growth, and Dual-Phase Hydration Modelling

**DOI:** 10.3390/plants15203131

**Published:** 2026-10-13

**Authors:** Tiwanat Gaewsondee, Phirayot Khaengkhan, Cherdpong Chiawchanwattana, Suphan Yangyuen

**Affiliations:** 1 Department of Mechanical Engineering, Faculty of Engineering, Nakhon Phanom University, Nakhon Phanom 48000, Thailand; tiwanat.g@gmail.com; 2 Department of Agricultural Technology, Faculty of Technology, Mahasarakham University, Maha Sarakham 44150, Thailand; perayos.k@msu.ac.th; 3 Department of Mechanical Engineering, Faculty of Engineering, Mahasarakham University, Maha Sarakham 44150, Thailand

**Keywords:** *Oryza sativa* L., seed ageing, magnetic seed treatment, imbibition kinetics, seed vigour, response-surface methodology, dual-phase Gompertz model

## Abstract

Seed ageing reduces germination and seedling performance, but variation in these responses and hydration kinetics across dynamic magnetic-field exposure (DMFE) conditions is poorly defined. Khao Dawk Mali 105 rice seeds (*Oryza sativa* L.) stored for 10–13 months were treated for 3, 6, or 9 min at nominal maximum surface flux densities of 150, 270, 370, or 470 mT. Untreated controls were excluded from the factorial analyses and used as descriptive references. Water uptake was modelled kinetically, and germination, vigour, and seedling growth by response surfaces; for early germination the arcsine-square-root scale was adopted after grouped cross-validation. Storage duration dominated every endpoint, especially early germination (*η*_p_^2^ = 0.991), declining approximately 28 percentage points per month. DMFE-associated differences were concentrated during early imbibition: treated seeds took up 2.9–4.9 percentage points more water than controls at 6 h, narrowing to 0.6–1.2 points by 42 h. The Reduced Dual-Phase Gompertz model was decisively favoured (ΔAICc = 260.2–347.2). Flux density was retained in the earlier hydration amplitude and all six seedling-growth models, and on the arcsine scale in a shallow early-germination profile whose selection was sensitive to design support and which did not improve cross-validated prediction. Independent seed lots, airflow-only controls, and field validation are required before practical recommendations.

## 1. Introduction

Seed vigour integrates germination speed, seedling emergence and growth, storability, and tolerance to environmental stress, and it, therefore, governs the uniformity of stand establishment [1,2]. Rice (*Oryza sativa* L., Poaceae) seed deteriorates progressively during storage, and elevated temperature and relative humidity accelerate the loss of vigour, germination performance, and subsequent seedling growth [1,3]. Germination speed commonly declines before final germination percentage does, so early germination and early seedling growth detect incipient ageing while germinability is still largely intact [1]. The problem is directly relevant to Khao Dawk Mali 105 (KDML 105), a widely cultivated aromatic cultivar in northeastern Thailand [4], whose germination performance changes across storage, from dormancy release to subsequent deterioration [5,6]. Physical seed-conditioning methods that improve the early performance of stored rice seed without leaving chemical residues are consequently of increasing interest [7,8].

Magnetic-field treatment is one such method and has been investigated as a non-chemical approach to seed and plant biostimulation [9]. Changes in germination rate and early seedling growth after magnetic exposure have been reported in rice [10,11], and treatment-dependent responses in germination, vigour, seedling elongation, and biomass have been reported in sunflower (*Helianthus annuus* L., Asteraceae), soybean (*Glycine max* (L.) Merr., Fabaceae), common bean (*Phaseolus vulgaris* L., Fabaceae), and barley (*Hordeum vulgare* L., Poaceae) [12,13,14,15]. Responses are not uniform; their direction and magnitude depend on magnetic flux density, exposure duration, seed orientation relative to the field, and seed physiological condition, and may also vary among species and genotypes [16,17,18,19]. They are also commonly non-monotonic, with higher performance at particular field–duration combinations rather than at the highest field strength or the longest exposure [12,16,18]. Proposed mechanisms include altered membrane permeability, ion and nutrient transport, respiration and enzyme activity, water relations, reactive-oxygen-species and antioxidant regulation, and hormone-related signalling [20,21,22,23], but the causal evidence remains incomplete and no single mechanism accounts for the responses across species and exposure systems. Magnetic treatment is, therefore, best regarded as a condition-dependent intervention rather than a universally beneficial priming treatment.

Most magnetic seed treatments have used static arrangements in which seeds remain fixed relative to the field source [10,17,24]. Because flux density varies spatially, seeds at different positions then receive unequal exposure, which limits treatment uniformity and reproducibility. Gaewsondee et al. [25] addressed this by developing an air-driven dynamic magnetic-field exposure (DMFE) system, in which vortex airflow continuously redistributes the seed batch and carries individual seeds repeatedly through a spatially non-uniform field, so that field–seed contact is broadened and persistent positional differences are reduced. Applied at 150 mT, that system produced exposure-duration-dependent changes in the vigour and early seedling growth of KDML 105 [25]. It was tested, however, at a single flux density and on relatively fresh, dormancy-released seed, so neither progressive storage deterioration nor the joint action of seed age, flux density, and exposure duration was addressed.

Where such a treatment might act is constrained by the physiology of imbibition. Early water entry rehydrates cellular structures and macromolecules, supports membrane reorganisation and repair, restores mitochondrial energy metabolism, and enables the reserve mobilisation required for radicle emergence [1,26]; these events occur along a time-dependent, multiphasic uptake trajectory rather than as a single endpoint [27]. In rice, soaking behaviour differs between the initial rapid-uptake and later equilibration periods [28,29,30,31], but how progressive storage deterioration alters that trajectory is less well characterised. Magnetic exposure has separately been associated with changes in plant water relations and membrane transport, although direct evidence linking it to early seed-water movement is limited [24,32]. Two consequences follow for measurement. A treatment that changes the timing rather than the extent of hydration may be weak or undetectable at the endpoint; and even with time-resolved data, a kinetic model that cannot separate an earlier from a later component will absorb such a treatment term into its own residual variance. Detectability, therefore, depends jointly on when the response is measured and on how much structure the fitted model can resolve. The same argument applies to the treatment responses themselves: factorial ANOVA tests differences among the discrete levels imposed, whereas response-surface methodology represents responses as continuous low-order functions of the same quantitative predictors [33], and the two need not agree.

What has not been established is whether treatment-associated differences that have substantially diminished by the hydration endpoint and no longer show a consistent ranking among treatment levels—and that are not retained by a single-component kinetic model—become identifiable once the trajectory is resolved into components—and whether the factor structure recovered in that way agrees with what a categorical analysis of the same seeds reports. This study, therefore, examined KDML 105 seed stored for 10–13 months in order to (i) quantify the effects of storage duration, magnetic flux density, and exposure duration on water uptake, germination, vigour, and early seedling growth; (ii) compare Logistic, Gompertz, and Reduced Dual-Phase Gompertz descriptions of the hydration trajectory under an information criterion that penalises the additional parameters, and identify which treatment terms each model structure is able to retain; and (iii) describe the same responses as continuous surfaces over the treated domain and compare the factor structure they recover with that reported by the factorial analysis. It was hypothesised that storage duration would impose the dominant negative gradient across all responses; that DMFE would act preferentially on early hydration and on rate-sensitive endpoints rather than on integrative ones; that responses would be non-monotonic in flux density and exposure duration; and that the dual-component structure would remain preferred after its additional parameters were penalised, and would retain treatment terms that the single-component alternatives cannot.

## 2. Results

### 2.1. Water Absorption During Imbibition

**Figure 1:**
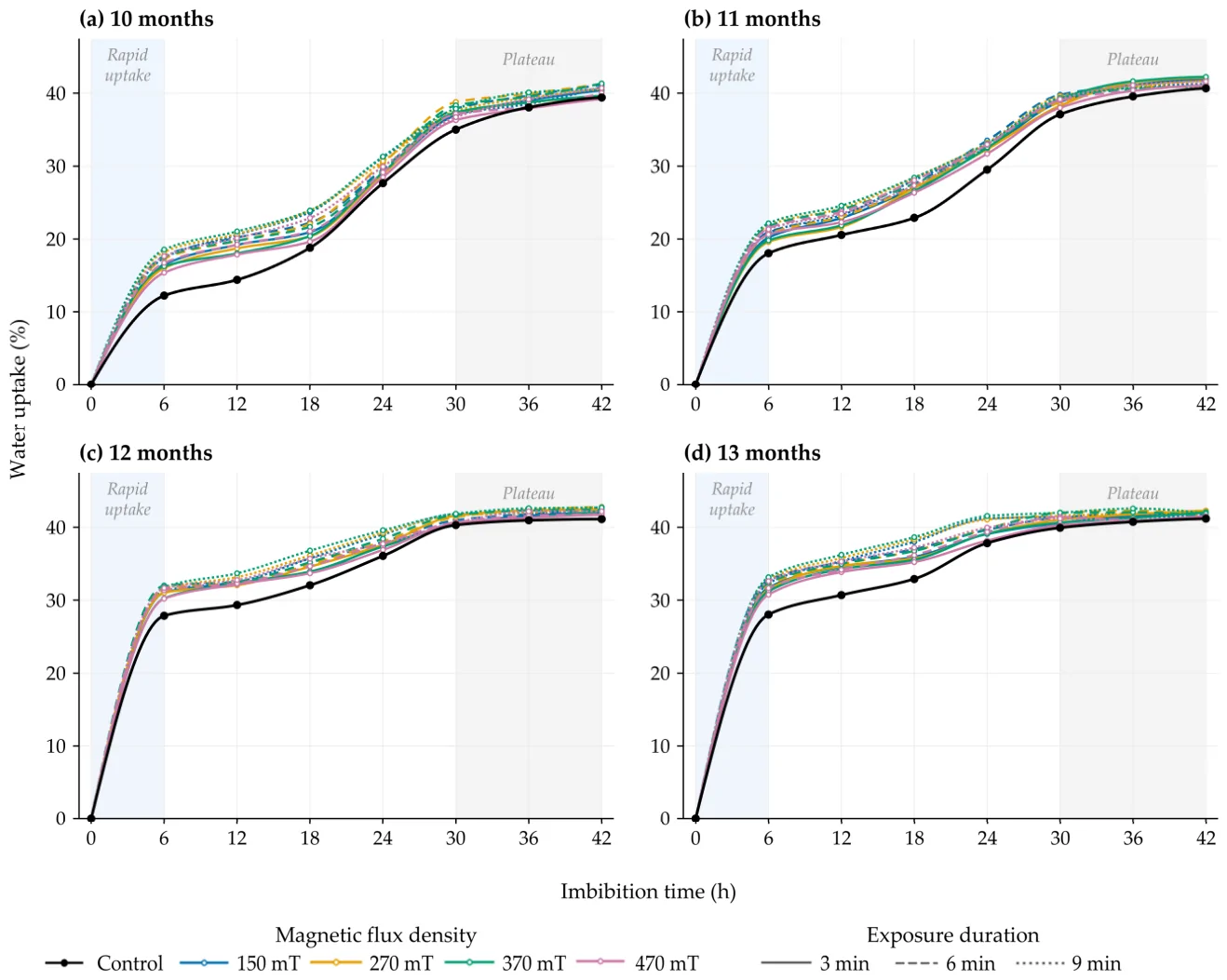
Water uptake of Khao Dawk Mali 105 (KDML 105) rice seeds (*Oryza sativa* L.) during 42 h of imbibition after (**a**) 10, (**b**) 11, (**c**) 12 and (**d**) 13 months of storage. Line colour indicates magnetic flux density, line style indicates exposure duration, and the black line with filled circles denotes the untreated control. Symbols mark the eight sampling times; curves are shape-preserving piecewise cubic Hermite interpolations between them and are not model fits. Shaded regions mark the rapid-uptake (0–6 h) and plateau (30–42 h) periods. Values are means of three independent replicates.

Water uptake increased rapidly during the first 6 h of imbibition, rose more gradually between 6 and 30 h, and approached a plateau from 30 to 42 h across all storage durations (Figure 1). DMFE-treated seeds generally showed higher mean water uptake than the untreated control during early imbibition, although the curves progressively converged as soaking continued. At 6 h, the mean treated–control difference ranged from 2.9 to 4.9 percentage points, while the largest treatment-specific difference within each storage-duration group ranged from 4.1 to 6.3 percentage points. For example, in the 10-month group, water uptake was 12.2% in the control and 18.5% after exposure to 370 mT for 9 min. The untreated control is the frame of reference against which these treated values are read. Because it received neither magnetic exposure nor vortex airflow, a treated–control difference cannot be attributed to the magnetic component alone. Control water uptake at every imbibition time, with the corresponding treated values, is given in Table S5.

The treated–control separation decreased to approximately 2.0–3.2 percentage points at 24 h and 0.6–1.2 percentage points at 42 h. At 42 h, water uptake ranged from 39.4% to 41.2% in the controls and reached up to 42.8% in the treated groups. No consistent ranking among magnetic flux densities or exposure durations was evident at this stage. Absolute water uptake also tended to increase with storage duration, with the older seed groups showing higher values during both early and final imbibition. This higher gravimetric uptake in the older seed groups does not necessarily indicate faster or more physiologically effective hydration.

### 2.2. Germination and Seed Vigour Responses

**Figure 2:**
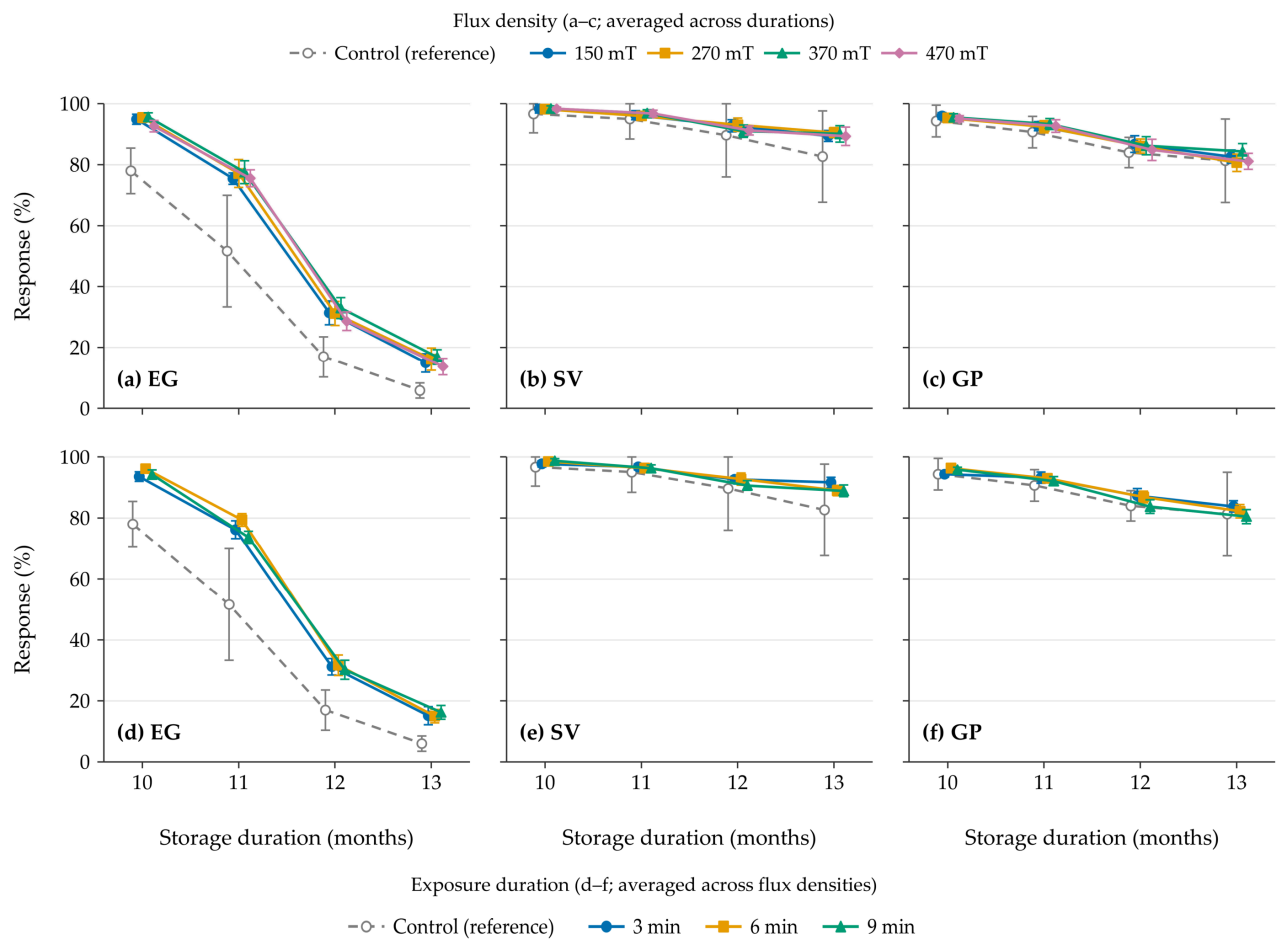
Marginal means of early germination (EG), seed vigour (SV) and final germination percentage (GP) in aged KDML 105 rice seeds. Panels (**a**–**c**), flux-density means averaged across exposure durations; panels (**d**–**f**), exposure duration means averaged across flux densities. Error bars are 95% confidence intervals (*n* = 9, 12 and 3 for the flux, duration and control series). The untreated control is a descriptive reference only.

Storage duration was the dominant source of variation in germination performance (Table 1; Figure 2). Across the 144 treated observations, early germination (EG), seed vigour (SV), and final germination percentage (GP) declined progressively from 10 to 13 months of storage. The storage-duration effect was greatest for EG, with a partial eta-squared of 0.991, followed by GP at 0.836 and SV at 0.785. This pattern indicates that the day-2 response was the most sensitive indicator of storage-related deterioration. Because SV records radicle emergence whereas GP counts only normally developed seedlings, the two measures are not nested, and SV exceeded GP in 138 of the 144 replicates. The normalised SV−GP gap (Section 4.4) rose from 2.9% at 10 months to 8.5% at 13 months (*F*(3, 96) = 30.35, *p* < 0.001, *η*_p_^2^ = 0.487). The underlying proportions are equal to or higher than the index, but the number of seeds not yet emerged at day five caps how much higher, and the increase between 10 and 13 months persists under the most conservative allocation: at most 4.6% at 10 months against at least 8.5% at 13 months.

Treatment effects were response-specific and substantially smaller than the effect of storage duration. Magnetic flux density significantly affected EG, with *p* = 0.007 and a partial eta-squared of 0.118, but did not significantly affect SV or GP. Exposure duration also affected EG and GP, with *p*-values of 0.034 and 0.008, respectively, whereas its main effect on SV was not significant. These patterns are reflected in Figure 2, where treatment-level separation was more evident for EG than for the later germination responses.

The storage duration × exposure duration interaction was significant for SV and GP, with *p*-values of 0.022 and 0.047 and partial eta-squared values of 0.140 and 0.122, respectively, indicating that exposure-duration responses varied across storage durations. The SV term is borderline, losing significance on the arcsine-square-root scale (*p* = 0.055) while the GP term strengthens (*p* = 0.024); it is nonetheless retained in the continuous SV model on both scales (Section 2.5). For EG the arcsine-square-root transformation changes no conclusion of this table: every effect keeps its significance status, and the flux-density main effect strengthens from *p* = 0.007 to *p* = 0.002. In contrast, the storage duration × magnetic flux density interaction, magnetic flux density × exposure duration interaction, and three-way interaction were not significant for EG, SV, or GP. The untreated control is the frame of reference against which the treated results are read, and Figure 2 shows it at each storage duration. It is excluded from the crossed factorial analysis for a structural reason rather than a substantive one: a single 0 mT, 0 min cell is not crossed with the flux-density and exposure-duration factors, so it contributes no degrees of freedom to any *B* or *T* effect and cannot enter a crossed factorial model. It is analysed instead by within-storage-duration Duncan comparison against every treated combination, reported in full in Table S5: the control is significantly the lowest for EG at all four storage durations, below the treated groups for SV only at 12 and 13 months, and not significantly different from most treated groups for GP at any storage duration. Because the control received neither magnetic exposure nor vortex airflow, these differences cannot be attributed to the magnetic component alone.

### 2.3. Seedling Growth Responses

Storage duration was the dominant source of variation in all six seedling-growth traits (Table 2). Its effect was largest for shoot dry weight, with a partial eta-squared of 0.918, followed by root length, root dry weight, root fresh weight, shoot fresh weight, and shoot length, with values ranging from 0.728 to 0.911. These results indicate a progressive decline in seedling elongation and biomass accumulation with increasing storage duration.

**Figure 3:**
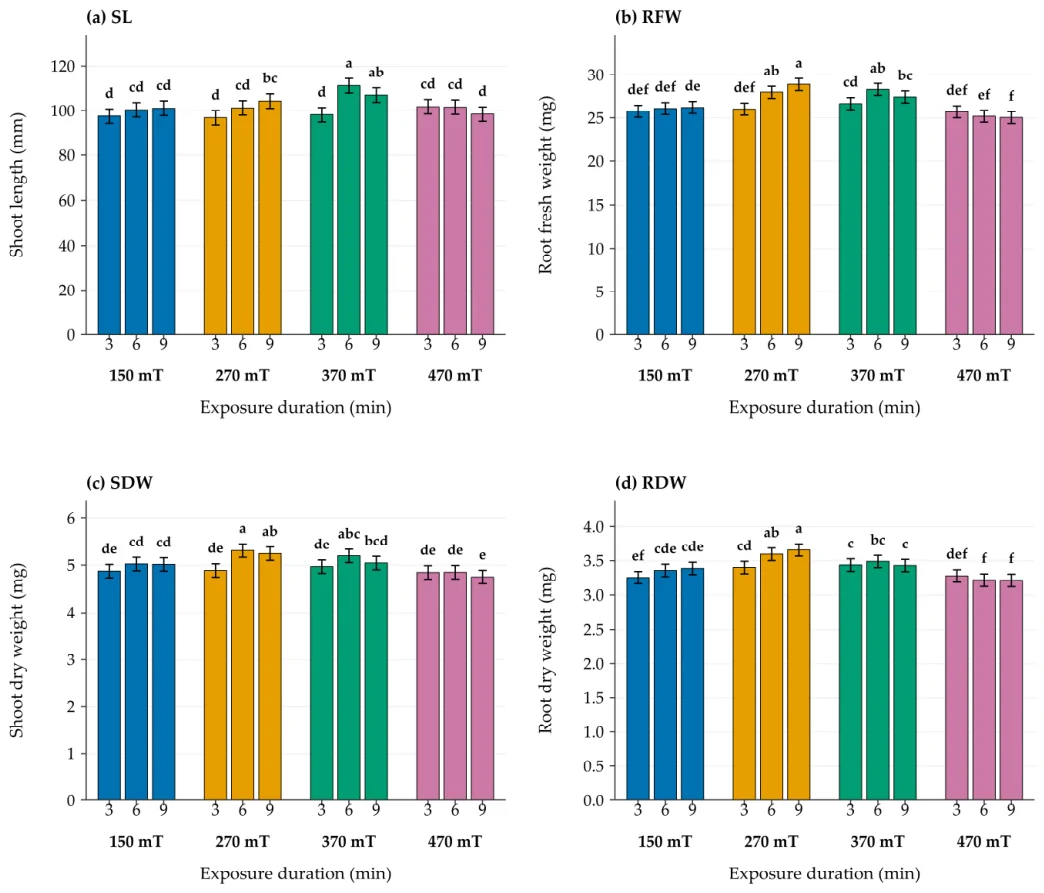
Magnetic flux density × exposure duration interactions for (**a**) shoot length, (**b**) root fresh weight, (**c**) shoot dry weight and (**d**) root dry weight of aged KDML 105 rice seeds, the four traits with a significant interaction. Bars are marginal means across the four storage durations (*n* = 12) and bar colour denotes flux density. Error bars are model-based 95% confidence intervals from the residual mean square of the three-way ANOVA (df = 96). Different letters indicate differences among the twelve combinations by Duncan’s multiple range test at *p* < 0.05. Weights are mg seedling^−1^.

Magnetic flux density and exposure duration also significantly affected all growth traits, although their effects were smaller than those of storage duration. Flux-density effects were strongest for root fresh weight, shoot fresh weight, and root dry weight. Exposure-duration effects were generally modest, with the smallest effect observed for root dry weight. For traits showing a significant magnetic flux density × exposure duration interaction—shoot length, root fresh weight, shoot dry weight, and root dry weight—comparatively higher marginal means occurred mainly at 270–370 mT combined with 6–9 min exposure (Figure 3). Responses did not increase monotonically with flux density: at 470 mT the marginal means ranked among the four lowest of the twelve combinations for root fresh weight, shoot dry weight, and root dry weight, and were intermediate for shoot length. Storage duration × magnetic flux density interactions were significant for root length and root fresh weight, whereas the storage duration × exposure duration interaction was significant only for root length. These results indicate greater storage-duration-dependent treatment sensitivity in root-related traits than in shoot traits. No significant three-way interaction was detected for any seedling-growth response.

### 2.4. Water-Uptake Kinetics and Model Performance

**Figure 4:**
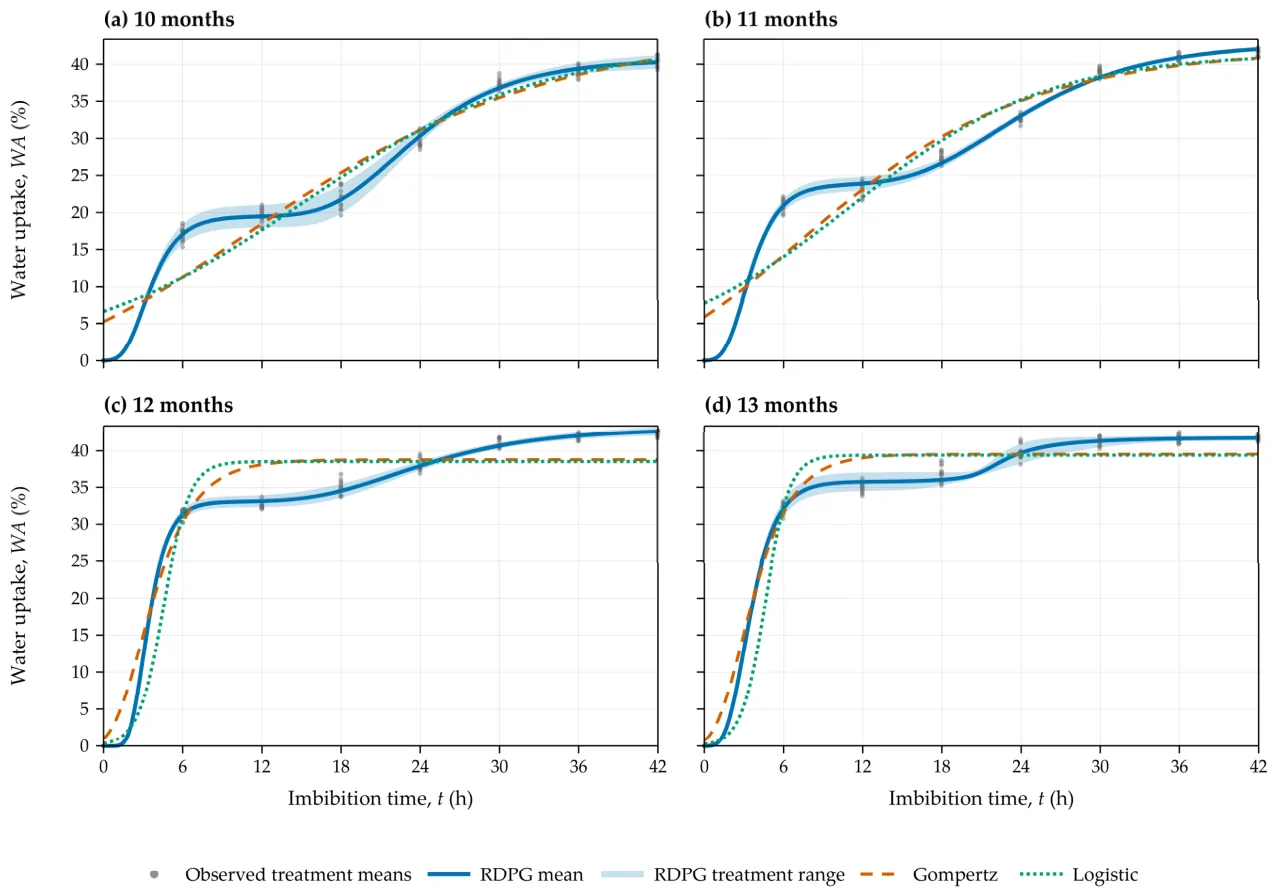
Observed and fitted water-uptake kinetics of KDML 105 rice seeds stored for (**a**) 10, (**b**) 11, (**c**) 12, and (**d**) 13 months. Grey symbols represent observed means for the 12 treated conditions. The solid line and shaded region show the mean and range, respectively, of the treatment-specific Reduced Dual-Phase Gompertz fits; dashed and dotted lines represent the Gompertz and Logistic fits. Untreated controls were excluded from model fitting.

Water-uptake kinetics were evaluated separately at each storage duration using 96 treated condition-by-time means, comprising 12 dynamic magnetic-field exposure combinations measured at eight imbibition times, with each mean calculated from three independent replicates. Untreated controls were excluded from model estimation and retained only as within-storage-duration descriptive references. Although all three models reproduced the overall time-dependent increase in water uptake, the Reduced Dual-Phase Gompertz (RDPG) model ranked first at every storage duration (Figure 4; Table 3). The RDPG yielded *R*^2^ values of 0.997–0.998, RMSE values of 0.51–0.66 percentage points, and MAE values of 0.38–0.50 percentage points, compared with *R*^2^ values of 0.908–0.970 and RMSE values of 2.27–3.95 percentage points for the single-component models. Despite its greater complexity (eight estimated coefficients versus three for each benchmark), the RDPG was strongly favoured, with ΔAICc values of 260.2–347.2 for the Logistic and Gompertz models.

The fitted trajectories illustrate the basis of this difference (Figure 4). The RDPG reproduced the intermediate shoulder between approximately 6 and 24 h, and the subsequent increase in water uptake, that the single-component models failed to capture adequately, and it continued to follow the observed rise beyond 24 h in the 12- and 13-month groups, where the Logistic and Gompertz curves approached their asymptotic levels prematurely near 39%. The unconstrained single-component formulations also produced positive fitted values at *t* = 0, ranging from approximately 5.3 to 7.7 percentage points after 10–11 months and 0.2 to 1.0 percentage points after 12–13 months. These non-zero fitted origins reflect the mathematical forms of the models and were not interpreted as measured water uptake or initial seed water content.

Under the selected common structure, neither single-component model retained a dependency on magnetic flux density (*B*) or exposure duration (*T*); each, therefore, comprised three coefficients that were constant with respect to the treatment factors within each storage-duration-specific fit and was retained as a parsimonious benchmark (Table S2). In contrast, the RDPG allowed the earlier-component amplitude *A*_1_ to depend on both *B* and *T*, retained a constant earlier-component rate coefficient *K*_1_, and allowed the later-component amplitude *A*_2_ and rate coefficient *K*_2_ to depend on *T*. The component-location anchors were fixed at *τ*_1_ = 3 h and *τ*_2_ = 22 h and were not estimated (Table 4). Their influence was examined by re-running the whole selection over a grid of *τ*_1_ from 2.0 to 6.0 h and *τ*_2_ from 18.0 to 26.0 h in 0.5 h steps at every storage duration (Figure S5; Table S6). The RDPG was decisively favoured, by more than 10 AICc units at every storage duration, in 136 of the 153 anchor pairs, comprising *τ*_1_ = 2.0–5.5 h with *τ*_2_ = 18–26 h, where the minimum ΔAICc was 117.2. The remaining 17 pairs all have *τ*_1_ = 6.0 h, where the RDPG remained preferred at 10 and 11 months but not at 12 and 13 months. The advantage of the dual-component representation is, therefore, robust across most of the tested anchor region and conditional at its upper boundary, where the earlier component becomes weakly identified because the rapid phase is represented by a single post-baseline observation, at 6 h. Longer exposure was associated with a larger earlier-component amplitude, with *A*_1*T*_ ranging from 0.204 to 0.391 percentage points min^−1^, whereas *A*_2*T*_ was negative at all storage durations (−0.014 to −0.406 percentage points min^−1^). These opposing directions indicate changes in the relative contributions of the two fitted components, while the net effect on their summed amplitude varied among storage durations.

The selected common RDPG structure also retained a negative magnetic-flux-density dependence in *A*_1_, with *A*_1*B*_ estimates ranging from −0.041 to −0.219 percentage points per 100 mT across storage durations (Table 4). Removing *B* from *A*_1_ increased the summed AICc by 20.7 units, and the *B*-containing structure won that two-model summed-AICc contest in 97.1% of 1000 condition-level cluster-bootstrap resamples. That figure is not a per-storage-duration selection frequency, and the two should not be read as comparable: when the structure is instead chosen afresh from all 256 candidates in each of 1000 resamples, *A*_1_ retains a *B* term in approximately 88% of resamples at 10 months and in 81.1%, 43.2% and 21.5% at 13, 12 and 11 months, respectively (Table S7). Storage-duration-specific evidence was correspondingly heterogeneous: approximate model-based confidence intervals excluded zero at 10 and 13 months, whereas the 95% condition-resampling percentile interval excluded zero only at 10 months, and then only narrowly (−0.4148 to −0.0103 over 10,000 resamples). Moreover, when structures were evaluated separately at 11 months, the formulation without *B* had an AICc approximately 2.0 units lower. The magnetic-flux-density term is therefore interpreted as a well-supported feature of the common dependency structure rather than as an effect established independently at every storage duration. Its negative sign describes fitted variation within the treated domain only and does not represent a comparison with the untreated control.

The RDPG ranked first at all four storage durations in 100% of the cluster-bootstrap resamples, while leave-one-treatment-condition-out cross-validation produced RMSE inflation factors of approximately 1.09–1.14, supporting predictive stability. The exposure-duration dependence of *K*_2_ was likewise stable where it was retained: its sign was preserved in 100.0%, 99.6% and 98.8% of 10,000 fixed-structure resamples at 10, 12 and 13 months, and a *T* term was retained in *K*_2_ in 97.3% of 1000 re-selections at 13 months, with a 95% Wilson interval of 96.1–98.1% (Table S7). At 12 months that retention depends heavily on one condition, *B* = 470 mT with *T* = 9 min, present in 89.6% against 49.3% of resamples without it, and this dependence is reported rather than smoothed over. Residual normality was acceptable in the 11- and 12-month groups but showed departures at 10 and 13 months (Shapiro–Wilk *p* = 0.012 and 0.021, respectively; Figures S1 and S2); coefficient-level intervals should, therefore, be interpreted cautiously. All fitted amplitudes and rate coefficients remained positive throughout the evaluated treatment domain.

### 2.5. Response-Surface Modelling of Germination Responses

Response-surface modelling was performed to describe the combined influence of storage duration, magnetic-field treatment conditions, and exposure duration on germination responses within the treated experimental region. The analysis used the 144 treated observations only. Starting from the full second-order model, reduced models were obtained under hierarchy-constrained backward elimination. Because the three responses are percentages, each was modelled on both the percentage and the arcsine-square-root scale, and the scale for each response was chosen by repeated grouped 8-fold cross-validation rather than by an information criterion, which cannot compare models fitted to different response variables. For early germination the arcsine-square-root scale predicted better and was adopted as primary; for seed vigour and final germination percentage it gave no gain and the percentage scale was retained (Table S8). The final equations and model-adequacy statistics are summarised in Table 5, panel (A) for early germination on the transformed scale and panel (B) for the two percentage-scale responses.

**Table 5:** Final reduced response-surface equations and model adequacy statistics for germination responses of aged KDML 105 rice seeds under treated-only dynamic magnetic-field exposure: (**A**) early germination on the arcsine-square-root scale and (**B**) seed vigour and final germination percentage on the percentage scale.

**(A) Early Germination, Arcsine-Square-Root Scale**								
**Response**	**Final Reduced Equation (Centred Predictors)**	***R*^2^**	**Adj. *R*^2^**	**RMSE**	**MAE**	**AIC**	***F*(6, 137)**	**Model *p***
EG (day 2)	y* = 0.857614 − 0.33021 *A*_c_ − 6.6731 × 10^−5^ *B*_c_ − 0.000519785 *T*_c_ + 0.0222939 *A*_c_^2^ − 1.24883 × 10^−6^ *B*_c_^2^ − 0.00295325 *T*_c_^2^	0.963	0.961	0.0728	0.0599	−331.7	590.36	<0.001
**(B) Seed Vigour and Final Germination Percentage, Percentage Scale**								
**Response**	**Final Reduced Equation**	***R*^2^**	**Adj. *R*^2^**	**RMSE**	**MAE**	**AIC**	**Model *p***	**Interpretation**
SV (day 5)	SV = 114.80 − 1.703*A* + 2.286*T* − 0.2139*AT*	0.721	0.715	2.12	1.65	633.7	<0.001	Moderate; storage-duration × exposure-duration response
GP (day 14)	GP = 125.01 − 2.978*A* + 2.968*T* − 0.2819*AT*	0.784	0.779	2.80	2.21	712.7	<0.001	Moderate; storage-duration × exposure-duration response

Notes: Panel (A) reports early germination on the arcsine-square-root scale and panel (B) reports seed vigour and final germination percentage on the percentage scale. In panel (A) the response is y* = arcsin √(EG/100) in radians and the equation is written in the centred predictors in which it was fitted, *A*_c_ = *A* − 11.5 months, *B*_c_ = *B* − 315 mT and *T*_c_ = *T* − 6 min; *R*^2^, adjusted *R*^2^, RMSE and MAE in panel (A) are therefore in radians and are not comparable with the percentage-point values of panel (B). Predictions were inverse-transformed to percentages for interpretation and for Figure 5a, where the descriptive in-sample RMSE and MAE were 6.00 and 4.68 percentage points; all transformed-scale fitted values remained within [0, π/2], so inverse transformation required no clipping. In panel (B), *A*, *B* and *T* are storage duration (months), magnetic flux density (mT) and exposure duration (min) and the equations are in natural units. The linear *B*_c_ and *T*_c_ terms in panel (A) were retained to preserve marginality because their quadratic terms were retained; their individual tests represent local slopes at the centring values, and the joint tests are *F*(2, 137) = 3.150, *p* = 0.046 for *B*_c_ + *B*_c_^2^ and *F*(2, 137) = 2.048, *p* = 0.133 for *T*_c_ + *T*_c_^2^. AIC is reported only to document model selection and is comparable only between models fitted to the same response on the same scale; it must not be compared across responses or scales. Model *p* is the overall *F*-test. SV and GP were interpreted cautiously because residual diagnostics indicated mild heteroscedasticity near the upper percentage bound (Breusch–Pagan *p* = 0.001 and 0.004). RMSE, root-mean-square error; MAE, mean absolute error; AIC, Akaike information criterion. Full coefficients, standard errors and term-level *p*-values: Table S9 for early germination and Table S3 for seed vigour and final germination percentage.

**Figure 5:**
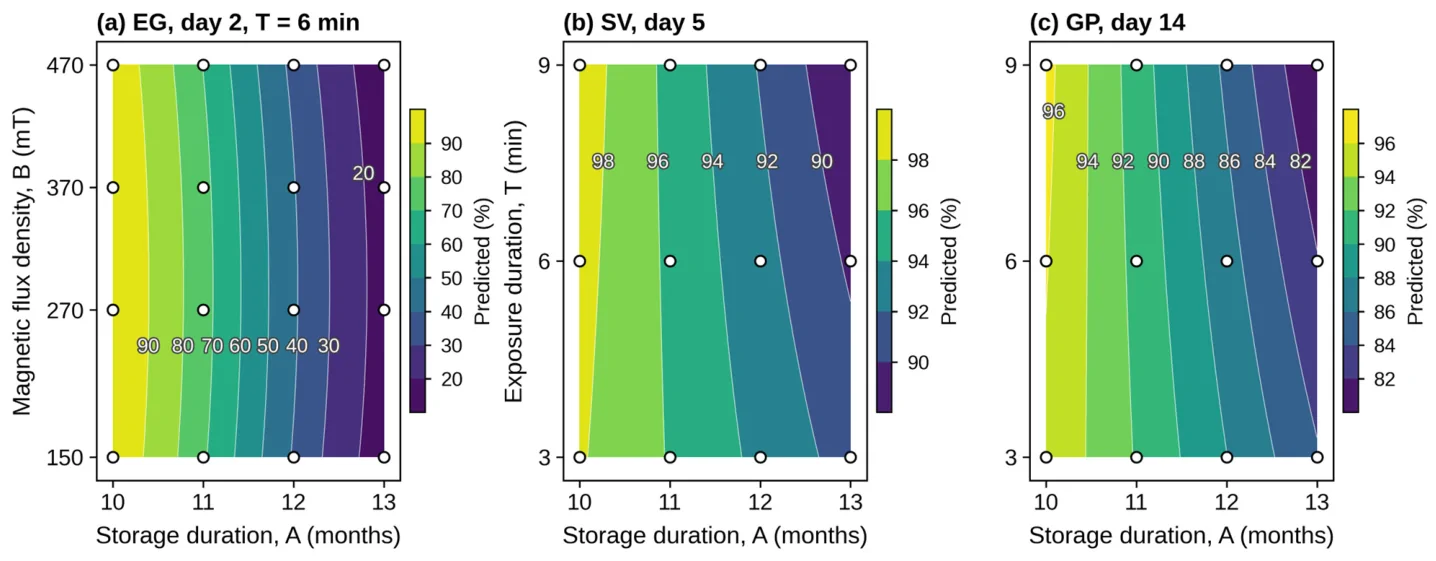
Predicted response surfaces for (**a**) early germination (EG) as a function of storage duration and magnetic flux density at an exposure duration of 6 min, and for (**b**) seed vigour (SV) and (**c**) final germination percentage (GP) as functions of storage duration and exposure duration. EG predictions were obtained on the arcsine-square-root scale and inverse-transformed to percentages; SV and GP were modelled directly as percentages. Open circles indicate the sampled *A* × *B* combinations in panel (**a**) and *A* × *T* combinations in panels (**b**,**c**). Colour scales are response-specific. Corresponding EG surfaces at exposure durations of 3, 6 and 9 min are shown in Figure S6.

For early germination (EG) the model selected by the published rule on the transformed response y* = arcsin √(EG/100) retained storage duration, magnetic flux density and exposure duration together with all three quadratic terms, *A*, *B*, *T*, *A*^2^, *B*^2^ and *T*^2^. The selection rule was not altered. Fit on the transformed scale was strong, with *R*^2^ = 0.963, adjusted *R*^2^ = 0.961 and RMSE = 0.0728 rad (Table 5A); after inverse transformation the descriptive in-sample RMSE and MAE were 6.00 and 4.68 percentage points. The scale was chosen on out-of-sample prediction, not on this in-sample fit: over 200 random grouped 8-fold partitions the arcsine pipeline gave a cross-validated RMSE of 6.651 ± 0.144 percentage points against 7.866 ± 0.153 for the percentage pipeline when flux-density terms were excluded from both, and 7.012 ± 0.204 against 8.107 ± 0.225 when they were allowed, the arcsine pipeline being better in 200 of 200 partitions in both comparisons (Table S8). The quoted spread is the standard deviation across the 200 overlapping partitions and is descriptive of fold-scheme variability; it is not a standard error and no confidence interval is attached to it. Because magnetic flux density is retained, Figure 5a shows predicted EG over storage duration and magnetic flux density with exposure duration fixed at 6 min; the corresponding surfaces at exposure durations of 3, 6 and 9 min are shown in Figure S6.

For seed vigour (SV), the final reduced model retained storage duration (*A*), exposure duration (*T*), and their interaction (*A* × *T*). The model provided a moderate fit to the treated-only data, with *R*^2^ = 0.721, adjusted *R*^2^ = 0.715, and RMSE = 2.12 (Table 5B). The corresponding contours in Figure 5b indicate that predicted SV remained high across the treated region but varied with the combined effect of storage duration and exposure duration. The interaction term shows that the predicted influence of exposure duration was not constant across storage durations.

A similar structure was retained for final germination percentage (GP), with *A*, *T*, and *A* × *T* included in the final reduced model. Model fit was moderate, with *R*^2^ = 0.784, adjusted *R*^2^ = 0.779, and RMSE = 2.80 (Table 5B). The predicted contours in Figure 5c show a gradual decline in GP with increasing storage duration, with exposure-duration effects expressed through the retained interaction. As with SV, the GP model was interpreted cautiously because residual diagnostics indicated mild heteroscedasticity near the upper percentage bound (Figures S3 and S4).

On the arcsine-square-root scale the early-germination model retained a quadratic flux-density profile comprising *B*_c_ and *B*_c_^2^, with the linear term retained under marginality, and magnetic flux density is therefore displayed in Figure 5a. Magnetic flux density was retained in neither of the two percentage-scale models, for seed vigour and final germination percentage, so it is not displayed in Figure 5b,c. The retained flux-density profile is shallow: across the tested range of 150–470 mT the predicted EG span at an exposure duration of 6 min is 1.50 percentage points at 10 months, 3.68 at 11 months, 3.99 at 12 months and 2.88 at 13 months. Its selection was sensitive to the available design support—retention of *B*_c_^2^ fell from 91.7% under leave-one-cell-out to 61.6% under repeated grouped 8-fold folds, and dropping any single flux-density level from the full data removed the term in three of the four cases (Table S10)—and allowing flux-density terms did not improve grouped cross-validated prediction, increasing RMSE in all 200 partitions on both scales. Overall, the response-surface models show that storage duration was the dominant predictor of germination performance, whereas exposure duration contributed to SV and GP through its interaction with storage duration and to EG through a quadratic term.

### 2.6. Response-Surface Modelling of Seedling-Growth Traits

Each seedling-growth trait was modelled within a continuous second-order response-surface framework using the 144 treated observations. Starting from the full quadratic model, terms were reduced by backward elimination under hierarchy and marginality constraints. The final equations and model-adequacy statistics are presented in Table 6, and the full coefficient estimates, standard errors, and term-level *p*-values are provided in Supplementary Table S4. All six reduced models were significant according to the overall *F*-tests (*p* < 0.001). Model explanatory performance ranged from *R*^2^ = 0.670 for shoot length (SL) to *R*^2^ = 0.898 for shoot dry weight (SDW). Adjusted *R*^2^ differed from *R*^2^ by 0.005–0.012 across models (Table 6). Residual assumptions were satisfactory for SL, RL, SFW, and SDW. The RFW model showed mild heteroscedasticity (Breusch–Pagan *p* = 0.004), whereas RDW showed a minor departure from residual normality (Shapiro–Wilk *p* = 0.027); the largest Cook’s distance in any model was below 0.13, and therefore below the conventional threshold of 1, and all leverage values were below 2*p*/*n*; no observation met these diagnostic criteria for undue influence.

Storage duration (*A*), magnetic flux density (*B*), and exposure duration (*T*) were present as lower-order terms in all six final models, with lower-order terms retained where required by model hierarchy. Across the tested region, predicted values of all six growth traits decreased with increasing storage duration. The RL and RFW models additionally retained *A*^2^, producing curved storage-duration profiles; nevertheless, their predicted responses remained decreasing throughout the experimental range of 10–13 months. Magnetic flux density was retained in every seedling-growth model, and the coefficient of *B*^2^ was negative for all six traits. Consequently, the fitted responses were concave across the tested flux-density range, with higher predicted values occurring at intermediate flux densities than at the lower and upper boundaries. The same concave form was retained for early germination on the arcsine-square-root scale, although far more weakly and with unstable selection (Section 2.5), and was retained in neither of the two percentage-scale germination models.

Exposure duration was included in all final models, with negative *T*^2^ terms for SL, RL, SFW, and SDW. The *A* × *T* interaction was retained for RL, SFW, and SDW and the *B* × *T* interaction for RFW, SDW, and RDW, so the fitted exposure-duration gradient varied with storage duration in the first group and with flux density in the second. SDW retained both, and no *A* × *B* interaction was retained for any trait (Table 6).

**Figure 6:**
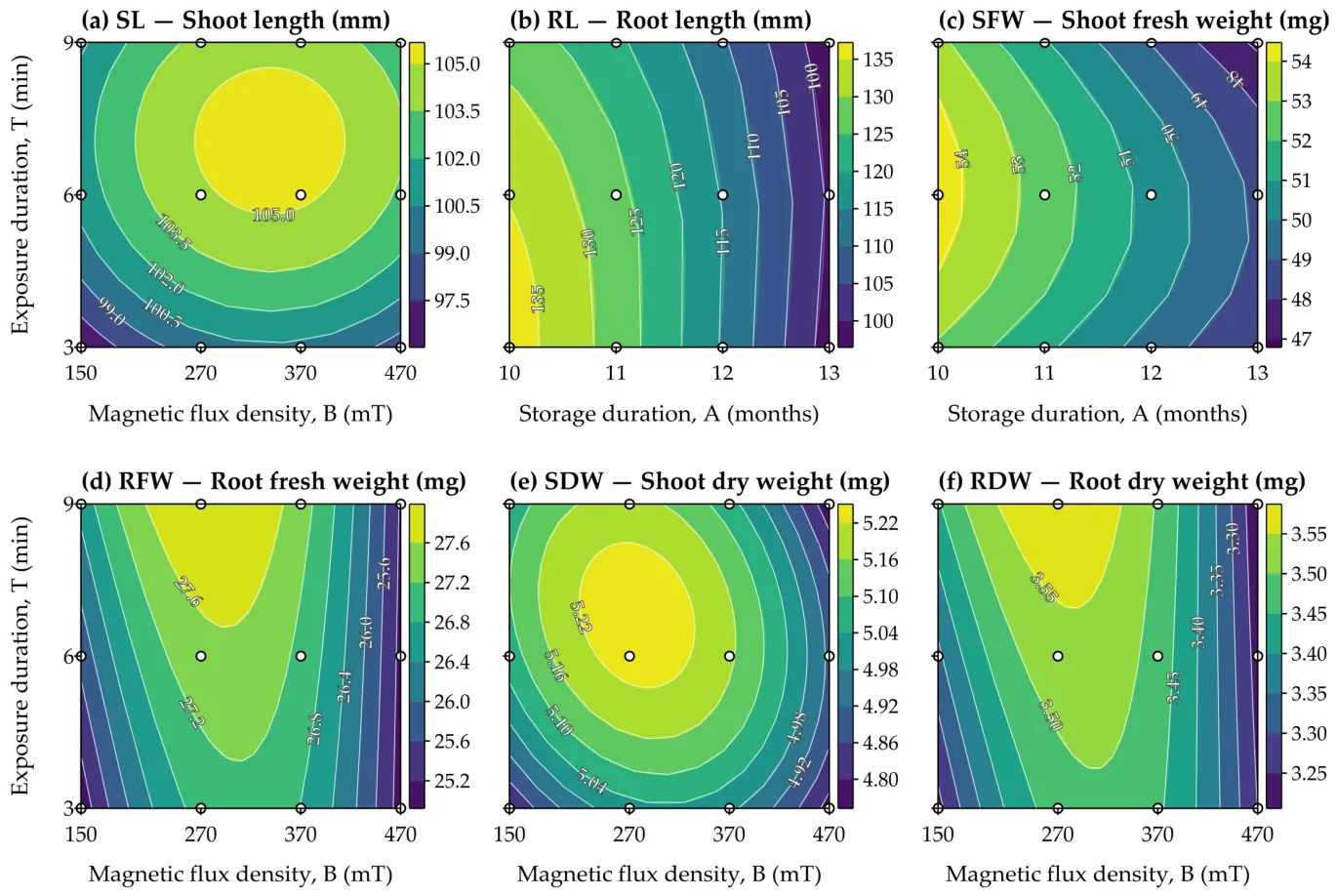
Predicted response contours for seedling-growth traits of aged KDML 105 rice seeds, from the final reduced models of Table 6: (**a**) shoot length (SL), (**d**) root fresh weight (RFW), (**e**) shoot dry weight (SDW) and (**f**) root dry weight (RDW) against magnetic flux density (*B*) and exposure duration (*T*) at *A* = 11.5 months; (**b**) root length (RL) and (**c**) shoot fresh weight (SFW) against storage duration (*A*) and *T* at *B* = 315 mT. Each trait is drawn in the plane of its retained interaction, or of its two quadratic terms where none was retained. Open circles mark projected design locations. Colour scales are response-specific; weights are mg seedling^−1^.

Figure 6 presents representative two-factor sections of the fitted models. Each trait is shown in the plane of its retained two-factor interaction; SL, which retained no interaction, is shown in the plane of its two quadratic terms. The fitted sections showed an interior curved region for SL, progressive storage-duration declines with exposure-duration gradients that varied across storage durations for RL and SFW, and curved response ridges for RFW, SDW, and RDW (Figure 6a–f). For visualisation, the unplotted predictor was held at its centred reference value: *A* = 11.5 months for the *B* × *T* panels and *B* = 315 mT for the *A* × *T* panels. These reference values were used only to obtain two-dimensional model sections and were not experimentally tested factor levels.

## 3. Discussion

Seed ageing set the performance ceiling: storage duration dominated every germination and seedling-growth endpoint. Differences associated with dynamic magnetic-field exposure (DMFE) were concentrated in a narrow window during early imbibition and faded as hydration approached equilibrium. Whether these differences were detectable, however, depended on the response class examined, on the measurement scale, and on the resolving power of the model applied—magnetic flux density was retained as a continuous term in the hydration and seedling-growth models and, on the arcsine-square-root scale, in the early-germination model as well, but in neither of the percentage-scale germination models.

### 3.1. Ageing Sets the Performance Ceiling

Storage duration established the strongest and most consistent gradient in the experiment. Early germination (EG), seed vigour (SV), and final germination percentage (GP) declined progressively from 10 to 13 months (*η*_p_^2^ = 0.991 for EG, 0.836 for GP, and 0.785 for SV), and the same dominance held for all six seedling-growth traits (*η*_p_^2^ = 0.728–0.918). The disproportionate response of EG separates the rate of germination from the capacity to complete it: early counts reflect how rapidly seeds resume coordinated metabolism after imbibition [1,2], whereas a day-14 assessment allows slower seeds additional time to produce normal seedlings [34]. Rapid, uniform germination depends on efficient membrane reorganisation, timely respiratory activation, enzyme synthesis, and reserve mobilisation—processes that deteriorate before germinability is fully lost [1,3,35]. The steeper decline in EG than in SV or GP is consistent with that sequence, although germination was recorded only at fixed day-2, day-5, and day-14 checkpoints, so germination synchrony was not directly quantified.

A second pattern is evident in the derived SV−GP bounds. The normalised SV−GP gap widened from 2.9% at 10 months to 8.5% at 13 months, and the widening holds even when every seed not yet emerged at day 5 is assumed to have become a normal seedling (Section 2.2). The widening bounds therefore indicate that longer storage was associated not only with slower germination but also with a greater shortfall between day-5 emergence and subsequent normal-seedling development, although the two processes cannot be resolved at the individual-seed level in the present design.

The continuous models reinforced this pattern. Storage duration dominated the early-germination response surface (Table 5A). Magnetic flux density and exposure duration were also retained on the arcsine-square-root scale, but their fitted variation over the tested ranges was small relative to the storage-duration gradient (Section 2.5). The percentage-scale comparator reported in Table S3 estimates a decline of approximately 28 percentage points per additional month over the tested 10–13-month interval. Because the primary Table 5A model is nonlinear after back-transformation to the percentage scale, this value should not be interpreted as a constant percentage-scale slope of the primary model. Storage duration also reduced elongation and biomass accumulation. Because growth traits were measured on normal seedlings at day 14, these reductions indicate diminished post-germination performance rather than simply fewer seeds germinating. Impaired reserve mobilisation, lower respiratory efficiency, membrane damage, oxidative injury, and disrupted antioxidant systems are frequently proposed components of seed deterioration [1,3,35], but none was measured here. Rice-specific evidence also links ageing tolerance with reduced H_2_O_2_ accumulation in the radicle [36]. The present results, therefore, establish the magnitude and endpoint sensitivity of deterioration without attributing it to a specific cellular mechanism.

### 3.2. DMFE-Associated Differences During Early Hydration

DMFE-treated seeds showed descriptively greater water uptake than the untreated control during early imbibition at every storage duration. At 6 h the mean treated–control difference was 2.9–4.9 percentage points, reaching 4.1–6.3 percentage points for the most responsive treatments, then narrowing substantially by 42 h, by which time no consistent ranking among flux densities or exposure durations remained (Figure 1). Because water uptake was evaluated descriptively rather than by an inferential repeated-measures analysis, these differences represent temporal trends rather than statistically confirmed effects. Their concentration in the early phase is consistent with an association with the timing of hydration rather than with final water-holding capacity. Across storage durations, greater early gravimetric water uptake coincided with markedly lower EG in the older seed groups, indicating that greater mass gain during imbibition did not correspond to better physiological performance.

This localisation is physiologically meaningful. Initial water entry rehydrates cellular structures and macromolecules, promotes membrane reorganisation, and initiates recovery of respiration and other processes required for germination [1,26,27]; early imbibition may, therefore, constitute a sensitive window in which modest changes in water entry influence the timing of metabolic activation. Magnetic fields have been proposed to affect membrane permeability, ion transport, and plant water relations [22,24,32], but these mechanisms were not measured here. Two constraints limit causal inference. Hydration and germination were not linked through individual-seed measurements or mediation analysis, so greater early water uptake cannot be identified as the cause of earlier germination; and because the untreated control received neither vortex airflow nor passage through the chamber, the trend cannot be attributed uniquely to the magnetic component. An airflow-only control is required to separate magnetic from mechanical or aeration effects.

### 3.3. Response-Class Dependence of the Flux-Density Effect

The clearest structural result of this study is that magnetic flux density behaved differently across the three classes of response. In the hydration model it was retained in the amplitude of the earlier component; in the response-surface models it was retained in all six seedling-growth traits, with negative quadratic coefficients producing concave profiles (Figure 6); and in the germination models its retention depended on the measurement scale, appearing in the early-germination model on the arcsine-square-root scale (Figure 5a) but in neither percentage-scale model. The factorial analysis detected a significant flux-density effect on EG on both scales. Exposure duration showed the complementary pattern, contributing to germination mainly through its interaction with storage duration for SV and GP and through a quadratic term for EG. Flux density, therefore, contributed a retained continuous term to hydration and to seedling growth robustly, and to early germination only weakly and conditionally on scale—a graded pattern that emerged only by integrating the factorial, response-surface, and kinetic analyses.

The apparent contradiction for EG is a matter of what each analysis asks, and three questions have to be separated. Factorial ANOVA tests whether the four imposed flux-density levels differ categorically, whereas response-surface modelling evaluates whether those differences can be represented by a low-order polynomial trend. Resampling and grouped cross-validation then assess selection stability and incremental predictive value. Differences among 150, 270, 370, and 470 mT may be non-monotonic, or small relative to the dominant storage-duration gradient, and therefore poorly represented by a low-order polynomial term. Model reduction was additionally performed under hierarchy and marginality constraints, so lower-order terms were retained whenever required by a retained quadratic or interaction term; the final equations should be read from their complete structure rather than from isolated term-level *p*-values [37]. The measurement scale contributes as well. The storage-duration means of EG span 15–95% across the tested storage durations, against an observed range of 8–99% over the 144 treated replicates, so its variance is strongly mean-dependent; on the variance-stabilised scale flux density and its quadratic are retained (Section 2.5), describing a concave profile with higher fitted values at intermediate flux densities, approximately 270–370 mT—the same form retained for all six seedling-growth traits, though far shallower. The exclusion of flux density from the percentage-scale EG surface should, therefore, be regarded as a scale-sensitive model-selection result rather than as evidence that no flux-density-related pattern exists; equally, its retention on the transformed scale is not evidence of a stable or predictively useful effect, since allowing flux-density terms increased cross-validated prediction error in all 200 partitions.

The evidence supporting the flux-density term differs in strength between the two classes where it was retained. In the seedling-growth models the effect was unambiguous, with flux density significant for all six traits and higher-performing combinations occurring mainly within 270–370 mT and 6–9 min, whereas 470 mT did not consistently produce the greatest response (Figure 3). Responses dependent on magnetic-field strength and/or exposure duration have also been reported in rice and other crops, although their forms differ among species, endpoints, and treatment protocols [10,16,18,38]. In a rice tissue-culture system, biomass responses across 14–343 mT were greater at lower-to-intermediate field levels than at the highest level [11], whereas triticale germination followed an approximately linear magnetic-time relationship over a much narrower field range [39]. In the present study, root traits showed greater treatment sensitivity than shoot traits, with larger flux-density effect sizes for root than shoot length, fresh weight, and dry weight, and with age-dependent interactions concentrated in root traits. Greater root sensitivity is not, however, a general property: root-specific responses have been reported in rice and *Arabidopsis* [40,41], whereas magnetic exposure altered the barley shoot proteome more than the root proteome [15], and organ-specific patterns in maize and soybean depended on species and endpoint [42]. In the hydration model the evidence was strong at the level of the common structure but uneven among storage durations: removing flux density from the earlier-component amplitude increased the summed AICc by 20.7 units and the term was retained in 97.1% of 1000 condition-level cluster-bootstrap resamples, yet the coefficient interval excluded zero at 10 and 13 months under model-based inference and only at 10 months under the condition-level bootstrap, with the flux-free structure marginally preferred at 11 months. The coefficient was negative at every storage duration (−0.041 to −0.219 percentage points per 100 mT); this describes fitted variation within the treated domain and is not a comparison with the untreated control, which lies outside both treatment-factor ranges. Neither region should be described as an optimum, since no formal optimisation or independent validation was performed.

A testable hypothesis follows from this structure. If DMFE acts principally on the earlier hydration component rather than on total water uptake, then three consequences are expected: treatment differences in water content should be largest during early imbibition and substantially diminished by 42 h, as observed; the treatment should advance the timing of metabolic reactivation—detectable as an earlier respiratory rise and earlier activity of antioxidant and reserve-mobilising enzymes during the first hours of imbibition—without altering the corresponding endpoint values; and rate-sensitive and integrative endpoints should show different treatment sensitivities, consistent with the stronger flux-density signal observed for EG than for GP. The second prediction requires time-resolved biochemical sampling during early imbibition, which the present design did not include. This hypothesis is offered as a direction for experimental testing rather than as a conclusion supported by the present data.

### 3.4. What the Dual-Component Model Adds, and Its Limits

The Logistic and Gompertz functions describe hydration with a single asymptote and one kinetic component. Both consequently missed the intermediate shoulder between approximately 6 and 24 h and approached their asymptotic levels prematurely near 39%, underestimating continued uptake in the 12- and 13-month groups (Figure 4). Resolving the trajectory into two additive components addressed both discrepancies, and the RDPG remained decisively preferred after penalisation for its additional parameters (Table 3). The result is consistent with the established multiphasic character of seed imbibition [27,28,31,43].

Under the structure selected by minimising the summed AICc, the earlier-component amplitude depended on both exposure duration and flux density, the later-component amplitude and rate coefficient on exposure duration, and the earlier-component rate coefficient was constant. Longer exposure was associated with a larger earlier amplitude (0.204–0.391 percentage points min^−1^) and a smaller later amplitude (−0.014 to −0.406 percentage points min^−1^), so exposure duration was expressed as a change in the relative contributions of the two components; the net effect on their summed amplitude varied among storage durations, and no consistent change in total uptake can be claimed. Fixing the component-location parameters at *τ*_1_ = 3 h and *τ*_2_ = 22 h anchored the terms near the observed early-uptake and later-transition regions and left four kinetic parameters (*A*_1_, *K*_1_, *A*_2_, *K*_2_) for treatment-dependency specification. These values are empirical modelling choices derived from the present trajectories, not universally defined physiological transition times, and their transferability requires evaluation with independent seed lots and sampling schedules.

The selected structure was stable under resampling: the model ranked first at all four storage durations in every cluster-bootstrap resample, and leave-one-treatment-condition-out cross-validation inflated RMSE by only 1.09–1.14. These analyses establish internal stability and are not a substitute for external validation. The model should therefore be read as a parsimonious empirical representation of a progressively changing hydration trajectory, not as evidence that imbibition comprises two discrete, physiologically independent processes. The components may reflect overlapping changes in tissue hydration, membrane reorganisation, metabolic reactivation, and reserve mobilisation [26], none of which was measured. The principal contribution is methodological: an exposure-resolved framework for describing water uptake within the tested 0–42 h interval without mechanistic claims beyond the evidence.

### 3.5. Implications, Limitations, and Validation

The practical scale of the treatment effect is best judged against the ageing gradient it operates within. EG fell by approximately 28 percentage points per additional month of storage (Section 3.1), whereas the span between the highest- and lowest-performing treatment combinations was about 5 percentage points averaged across storage durations and 6–9 percentage points within individual storage durations. The treatment-associated span was, therefore, small relative to the ageing gradient, indicating that treatment-related variation occurred within, rather than reversed, the deterioration trajectory. The fitted optima also differ among traits, from approximately 240 mT for root length to approximately 339 mT for shoot length and shoot dry weight, so no single flux–duration setting is identified by these data; a formal multi-response optimisation was not performed, and one computed from six surfaces fitted to a single seed lot without external validation would imply a precision the design cannot support. The guidance these results do support is directional: the higher fitted values for the responding traits lie within 270–370 mT and 6–9 min, and the choice among combinations in that region is small relative to the effect of seed age. As a dry, non-chemical treatment, DMFE may hold practical value where rapid seed conditioning is preferred [7,8,44], but these results were obtained under controlled laboratory conditions and establish no benefit for field emergence, crop establishment, grain yield, throughput, or commercial scalability.

Several limitations restrict generalisation. The study used one cultivar, a single harvest lot, one exposure apparatus, and a limited range of storage durations and treatment conditions. The untreated control received neither magnetic exposure nor vortex airflow, so magnetic effects cannot be separated from mechanical, aeration, or chamber-passage effects. Individual seed trajectories and position-dependent magnetic exposure histories were not quantified. Water uptake was evaluated descriptively rather than inferentially. Only the factorial ANOVAs of the germination and seedling-growth responses are primary inferential analyses; the model-selection, scale-comparison, sensitivity and resampling results are exploratory (Section 4.7), and no finding is confirmatory beyond the tested cultivar, harvest lot and treatment range. No biochemical or molecular measurements were made that could test the proposed links among DMFE, membrane reorganisation, oxidative status, respiration, and reserve mobilisation. Residual diagnostics were generally acceptable, but the hydration model departed from residual normality at 10 and 13 months (Shapiro–Wilk *p* = 0.012 and 0.021), and among the growth models the root fresh weight model showed mild heteroscedasticity and the root dry weight model a minor departure from normality; coefficient-level intervals should be read accordingly. Predictions apply only within *A* = 10–13 months, *B* = 150–470 mT, *T* = 3–9 min, and *t* = 0–42 h, and must not be extrapolated to the untreated control.

Future work should evaluate independent harvest lots and additional cultivars, incorporate sham and airflow-only controls, and test whether laboratory responses translate into field emergence and early establishment. Mechanistic studies should target the predictions set out in Section 3.3, with time-resolved measurement of membrane integrity, respiration, antioxidant status, and reserve-mobilising enzymes during the first hours of imbibition. The kinetic model should be tested against independently sampled trajectories with denser early-time measurements and with component-location parameters estimated rather than fixed where the data allow. An external validation dataset and a formally designed optimisation study would be required before any reproducible treatment window could be recommended. Within these limitations, this study provides a quantitative basis for the further development of DMFE as a seed-conditioning technology rather than a validated agronomic recommendation.

## 4. Materials and Methods

### 4.1. Rice Seed Material and Storage Conditions

Seeds of Khao Dawk Mali 105 rice (*Oryza sativa* L., Poaceae; cv. KDML 105) were obtained from Nakhon Phanom Province, Thailand. All seed material used in the study originated from a single harvest lot. Following harvest, the seeds were sun-dried before storage to approximately 14% moisture content on a wet basis, as determined using the low-constant-temperature oven method specified in the International Rules for Seed Testing [34]. Foreign materials and visibly damaged or morphologically abnormal seeds were removed before storage. The cleaned seeds were placed in sealed plastic containers and stored under ambient laboratory conditions at 25–28 °C and 50–80% relative humidity. Treatment batches were defined gravimetrically rather than by a fixed seed number. Based on the measured thousand-seed weights, which ranged from 23.785 to 24.959 g across the 10–13-month storage groups, each 100 g treatment batch was estimated to contain approximately 4007–4204 seeds, whereas each 50 g sample used for the hydration experiment was estimated to contain approximately 2003–2102 seeds. Subsamples were withdrawn from the same stored lot after 10, 11, 12, and 13 months to establish the four storage-duration groups. Seed moisture content was measured at each withdrawal and ranged from 11.5 to 12.2% (wet basis). At each withdrawal, the subsamples were immediately subjected to treatment and response evaluation. This single-lot sampling design minimised variation attributable to harvest lot and enabled storage duration to represent elapsed ageing time across the experiment. All storage-duration groups were subsequently subjected to the same treatment allocation and response-evaluation procedures.

### 4.2. Dynamic Magnetic-Field Exposure Apparatus and Treatment Conditions

Dynamic magnetic-field exposure (DMFE) was applied using an air-driven apparatus adapted from Gaewsondee et al. [25]. The apparatus comprised a transparent vertical cylindrical chamber (outer diameter, 100 mm; inner diameter, 92 mm; height, 150 mm) positioned above an assembly of neodymium–iron–boron permanent magnets. For each treatment, a 100 g seed batch was circulated continuously within the chamber by vortex airflow supplied through the chamber base at a constant gauge pressure of 4 bar, verified before every treatment. Seed movement resulted in repeated traversal through spatially varying regions of the magnetic field. Four magnet configurations were used; the maximum magnetic flux densities at the magnet surface were 150, 270, 370, and 470 mT, measured with a digital magnetometer (Tenmars TM-197, Tenmars Electronics Co., Taipei, Taiwan). These surface measurements defined the nominal treatment levels. Because the seeds circulated continuously through a spatially non-uniform stationary field, the instantaneous flux density experienced by an individual seed varied with its position and was not measured directly. Seed batches were exposed for 3, 6, or 9 min. The term dynamic exposure, therefore, refers to continuous seed movement through the spatial gradient of a stationary permanent-magnet field rather than to temporal modulation of the magnetic source.

### 4.3. Experimental Design and Response Variables

The experiment followed a fully crossed 4 × 4 × 3 factorial design comprising storage duration (*A*: 10, 11, 12, and 13 months), magnetic flux density (*B*: nominal maximum 150, 270, 370, and 470 mT), and exposure duration (*T*: 3, 6, and 9 min), yielding 48 treated combinations. Every flux density was therefore tested at every exposure duration and at every storage duration. The three exposure durations were set at 3, 6 and 9 min to bracket the 6 min condition at which the same apparatus produced exposure-duration-dependent responses in KDML 105 in the earlier study [25], with equal spacing so that linear and quadratic exposure-duration terms could be estimated from three levels. Each combination comprised three independently treated 100 g seed batches, approximately 4007–4204 seeds each (Section 4.1), giving 144 treated experimental units. The three batches were maintained as independent replicates and were not pooled before response evaluation. An untreated control (0 mT, 0 min; no chamber or vortex-airflow exposure) was included at each storage duration and serves as the frame of reference for the treated results. It is not crossed with the flux-density and exposure-duration factors, so it contributes no degrees of freedom to any *B* or *T* effect and cannot enter the crossed factorial ANOVA or the response-surface models; it is analysed instead by within-storage-duration Duncan comparison against every treated combination (Table S5). Because it received neither magnetic exposure nor vortex airflow, control–treated differences confound the magnetic component with mechanical, aeration and chamber-passage effects. Measured responses were water uptake during imbibition (WA); early germination (EG, day 2), seed vigour (SV, day 5), and final germination percentage (GP, day 14); and day-14 shoot length (SL), root length (RL), shoot fresh weight (SFW), root fresh weight (RFW), shoot dry weight (SDW), and root dry weight (RDW). Measurement procedures are described in Section 4.4 and Section 4.5.

### 4.4. Germination and Seedling Evaluation

Germination was evaluated using the between-paper method. For each independent replicate, 100 seeds were placed between moistened paper sheets and incubated at 25 ± 2 °C for 14 days. Cumulative radicle emergence, defined as a radicle length of at least 2 mm, was recorded on days 2 and 5 and expressed as early germination (EG) and seed vigour (SV), respectively. Final germination percentage (GP) was determined on day 14 as the percentage of normal seedlings according to the International Rules for Seed Testing [34]. EG and SV therefore record radicle emergence, whereas GP counts only normally developed seedlings, excluding decayed and abnormal ones; the criteria are not nested, so SV may exceed GP. Because SV and GP are aggregate counts rather than individually tracked seed outcomes, an exploratory interval was derived for the proportion of seeds that had emerged by day 5 but did not become normal seedlings by day 14; the lower and upper bounds were computed for each replicate as

*L* = max(0, SV − GP)/SV,   *U* = [(SV − GP) + min(100 − SV, GP)]/SV

where SV and GP are percentages of the same 100-seed replicate. The resulting bounds were multiplied by 100, reported as percentages, and averaged within each storage-duration group [45]; *L* is reported as the normalised SV−GP gap.

On day 14, 70 normal seedlings were randomly selected from each replicate for measurement of shoot length (SL), root length (RL), shoot fresh weight (SFW), root fresh weight (RFW), shoot dry weight (SDW), and root dry weight (RDW). Shoot length was measured from the seed base to the shoot tip, and root length from the seed base to the tip of the longest root. Shoots and roots were separated and weighed immediately on a digital balance (GF-1000, A&D Company, Ltd., Tokyo, Japan), oven-dried at 80 °C for 48 h, and reweighed to determine dry weight. Shoot and root lengths were recorded for each of the 70 seedlings and averaged within the replicate, whereas the separated shoots and roots were weighed in bulk for each replicate and the totals divided by 70; fresh and dry weights are, therefore, expressed per seedling. Each replicate contributed a single value per trait, and these 144 replicate-level values are the units of analysis.

### 4.5. Water Uptake Measurement

Water uptake (WA) was measured gravimetrically at every storage duration, for all treated combinations and the untreated control, using three independent 50 g seed samples per condition, approximately 2003–2102 seeds each (Section 4.1), one from each replicate batch. Each sample was immersed in 300 mL of distilled water at 25 ± 3 °C and weighed at 0, 6, 12, 18, 24, 30, 36, and 42 h. At each interval, the seeds were removed, gently blotted with absorbent paper, weighed to the nearest 0.001 g on the same balance, and returned to the soaking medium. The same samples were monitored throughout the 42 h imbibition period.

Water absorption at time *t* was calculated as:

(1) $$\mathrm{WA}(t)\;=\frac{W_{t}-W_{0}}{W_{0}}\times 100$$

where *WA*(*t*) is water uptake (%), *W_t_* is seed mass at time *t*, and *W*_0_ is the initial seed mass before imbibition.

### 4.6. Mathematical Modelling

Water-uptake kinetics were modelled separately for each storage duration using 96 treated condition-by-time means (12 flux-density × exposure-duration combinations at eight imbibition times), each from three independent replicates. The untreated control was excluded from model estimation because it is not crossed with the treatment factors and so supplies no information about any treatment-dependent coefficient; its trajectories are reported alongside the treated ones as a within-storage-duration descriptive reference (Figure 1; Table S5). The Logistic and Gompertz functions were used as single-component benchmarks, with the Gompertz formulation following [46]. To represent the multiphasic hydration trajectory, a Reduced Dual-Phase Gompertz (RDPG) model was formulated for the present study, and the three model structures were compared. The Logistic model was expressed as

(2) $$\mathrm{WA}\left(t\right)=\frac{A}{1+\exp\left[-k\left(t-t_{0}\right)\right]}$$

where *A* is the asymptotic water uptake, *k* the rate coefficient, and *t*_0_ the inflexion time. The Gompertz model was expressed as

*WA*(*t*) = *A*exp{−exp[−*k*(*t* − *t_i_*)]} (3)

where *t_i_* is the inflexion time. To represent the multiphasic hydration trajectory, the RDPG model was formulated as

*WA*(*t*) = *A*_1_exp{−exp[−*K*_1_(*t* − *τ*_1_)]} + *A*_2_exp{−exp[−*K*_2_(*t* − *τ*_2_)]} (4)

where *A*_1_ and *A*_2_ are the amplitudes of two additive mathematical components, *K*_1_ and *K*_2_ the corresponding rate coefficients, and *τ*_1_ and *τ*_2_ component-location parameters. The location parameters were fixed at 3 and 22 h before fitting and were not estimated, leaving four kinetic parameters (*A*_1_, *K*_1_, *A*_2_, *K*_2_) for treatment-dependency specification. The components were not assumed to represent physiologically independent processes.

For every model and storage duration, all coefficients were estimated jointly in a one-stage nonlinear least-squares fit, so that the same residuals were used for estimation and performance assessment. Rather than imposing a predefined structure, each estimable kinetic parameter was allowed to be constant, linear in exposure duration, linear in magnetic flux density, or additively linear in both:

*θ* = *γ*_0_ + *γ_B_B*_c_ + *γ_T_T*_c_ (5)

where *θ* denotes the kinetic parameter concerned and *B*_c_ = (*B* − 315)/100 and *T*_c_ = *T* − 6 are centred predictors, with the *B*_c_ and/or *T*_c_ terms omitted for the simpler candidate forms; no interaction or quadratic treatment term was included. Evaluating all combinations gave 64 candidate structures for each single-component model and 256 for the RDPG. A single common structure per model family was selected by minimising the sum of the storage-duration-specific AICc values, whereas the coefficients were estimated separately for each storage duration. Optimisation used a deterministic multistart search, retaining the minimum-residual solution.

Response-surface models were developed for all nine germination and seedling-growth responses defined in Section 4.3, using the 144 treated replicate-level observations. The full second-order polynomial was

*Y* = *β*_0_ + *β*_1_*A* + *β*_2_*B* + *β*_3_*T* + *β*_11_*A*^2^ + *β*_22_*B*^2^ + *β*_33_*T*^2^ + *β*_12_*AB* + *β*_13_*AT* + *β*_23_*BT* (6)

where *Y* is the predicted response and *A*, *B*, and *T* are storage duration, magnetic flux density, and exposure duration in natural units. The models were fitted by ordinary least squares with the predictors centred at 11.5 months, 315 mT, and 6 min to limit collinearity among the polynomial terms, and reduced by backward elimination at α = 0.05 under hierarchy and marginality constraints. Because the three germination responses are percentages, each was modelled both untransformed and as y* = arcsin √(*y*/100) in radians, and the scale for each response was chosen by repeated grouped 8-fold cross-validation over 200 random partitions. Folds were grouped by the 48 *A* × *B* × *T* cells, so that the three replicates of a condition always fell in the same fold; backward selection was re-run inside every training fold; arcsine predictions were inverse-transformed to percentages before any error was computed; RMSE on the percentage scale was the primary criterion, with MAE secondary; and both scales saw the same partitions, so every comparison is paired. The spread quoted across partitions is a descriptive standard deviation, not a standard error and not a confidence interval. An information criterion was not used for this choice, because the two models are fitted to different response variables and their likelihoods are not comparable without a Jacobian adjustment. On this criterion the arcsine-square-root scale was adopted for early germination and the percentage scale was retained for seed vigour and final germination percentage; the six seedling-growth traits were modelled untransformed throughout. Fold counts, seeds, the leave-one-cell-out variant, the cluster bootstrap and the associated diagnostics are reported in the Supplementary Materials. Equations are expressed in natural units for reporting, except the early-germination equation of Table 5A, which is given in centred predictors to match the fitted model. Performance was evaluated using *R*^2^, adjusted *R*^2^, RMSE, MAE, AIC, overall model significance, and the residual diagnostics described in Section 4.7.

Hydration-model performance was evaluated using *R*^2^, RMSE, MAE and RSS, and by information criteria computed with *n* = 96 and *K* = *p* + 1, where *p* is the number of independently estimated mean-model coefficients and the additional parameter represents residual variance: AIC = *n* ln(RSS/*n*) + 2*K*, AICc = AIC + 2*K*(*K* + 1)/(*n* – *K* − 1), and BIC = *n* ln(RSS/*n*) + *K* ln(*n*). The common Gaussian additive constant was omitted, which does not affect criterion differences or ranking. AICc was the primary criterion for structure selection and model ranking, with AIC and BIC as secondary measures [47,48]. Approximate 95% confidence intervals came from the nonlinear least-squares covariance approximation, and residuals were assessed graphically and by the Shapiro–Wilk test [49].

Because the eight observations within a condition are repeated measurements of one trajectory, the independent Gaussian-error assumption is an approximation, and AICc was used for comparative model selection rather than absolute likelihood inference. Robustness was assessed by 1000 condition-level cluster-bootstrap resamples, each resampled condition retaining its complete eight-point trajectory, and by leave-one-treatment-condition-out cross-validation [50]. Predictions were restricted to 0–42 h for imbibition time, 10–13 months for storage duration, 150–470 mT for magnetic flux density, and 3–9 min for exposure duration, and the treated-only hydration equations were not extrapolated to the untreated control.

### 4.7. Statistical Analysis

Germination and seedling-growth responses were analysed by three-way factorial ANOVA with storage duration (*A*), magnetic flux density (*B*) and exposure duration (*T*) as fixed factors, on the 144 treated replicate-level observations (48 combinations × three replicates; residual df = 96), using Type II sums of squares, which the balanced design makes identical to Type III. Untreated controls were excluded from this analysis because the single 0 mT, 0 min cell is not crossed with the *B* and *T* factors; they were compared with the treated groups by within-storage-duration Duncan’s test instead (Table S5). Effect sizes were partial eta-squared (*η*_p_^2^); significant effects were separated by Duncan’s multiple range test at α = 0.05 [51], with protection level αr = 1 − (1 − 0.05)r − 1 over r ordered means. The normalised SV−GP gap was evaluated as an exploratory derived response within the same factorial framework; it was not one of the response variables specified in advance. ANOVA assumptions were assessed with the Shapiro–Wilk test and Levene’s median-centred test [49,52]; response-surface diagnostics additionally comprised the Breusch–Pagan test, Cook’s distance and leverage against 2*p*/*n* [53,54]. Because the germination responses are percentages, both analyses were repeated on arcsine-square-root-transformed values; conclusions not preserved are identified in Section 2. Raw water-uptake trajectories were summarised descriptively; hydration kinetics were modelled as described in Section 4.6, without inferential repeated-measures analysis. Analyses used Python 3.10 (statsmodels 0.14, SciPy 1.15, NumPy 2.2), with Duncan’s test implemented from the studentized-range distribution. Analysis scripts implementing the model fitting, backward elimination and Duncan’s test are available from the corresponding author on request. During the preparation of this manuscript, the authors used OpenAI ChatGPT (GPT-5.6 Sol) and Anthropic Claude (Claude Opus 5) to assist with language refinement, cross-document consistency checking, and statistical coding. No experimental data were generated by these tools. All AI-assisted outputs were reviewed and validated by the authors.

The three-way factorial ANOVAs of the germination and seedling-growth responses specified in Section 4.3 are the primary design-based inferential analyses of this study, and their inferences apply to the 144 treated experimental units tested. The response-surface model selection, the hydration-model structure selection, the comparison of the percentage and arcsine-square-root scales for early germination, the component-location anchor grid, the cluster-bootstrap and model-reselection analyses, the leave-one-cell-out and cross-validation diagnostics, the fitted optima and the derived SV−GP gap are exploratory or robustness analyses, and their results are reported as such. Comparisons with the untreated control, including the within-storage-duration Duncan comparisons in Table S5, are descriptive, because the control is not crossed with flux density and exposure duration. Because the study used one cultivar and a single harvest lot without an external validation dataset, none of these findings constitutes confirmatory evidence beyond the tested experimental domain.

## 5. Conclusions

Storage duration was the dominant determinant of performance in aged KDML 105 rice seed. Early germination (EG), final germination percentage, and seed vigour all declined between 10 and 13 months (*η*_p_^2^ = 0.991, 0.836, and 0.785), as did all six seedling-growth traits, with EG falling by approximately 28 percentage points per additional month. DMFE-associated variation occurred within, but did not reverse, this ageing gradient.

Treatment-associated differences were concentrated during early imbibition, narrowing from 2.9 to 4.9 percentage points at 6 h to 0.6–1.2 percentage points by 42 h. The Reduced Dual-Phase Gompertz representation remained decisively favoured after its additional parameters were penalised, across most of the tested range of component-location anchors. Flux density was retained in the earlier hydration amplitude and in all six seedling-growth models. On the arcsine-square-root scale, the early-germination model retained a shallow quadratic flux-density profile; however, selection of this profile was sensitive to design support, and allowing flux-density terms did not improve grouped cross-validated prediction, so storage duration remained the dominant determinant. This graded pattern emerged only by integrating the factorial, response-surface, and kinetic analyses. Higher predicted seedling performance occurred at intermediate flux densities and longer exposures, but these are not optimised conditions, since no formal optimisation or independent validation was performed.

The methodological contribution is a treatment-resolved kinetic framework in which treatment dependence in hydration was identifiable, whereas the single-component alternatives retained none. Because no mechanistic measurements were made, and because the untreated control received neither magnetic exposure nor vortex airflow, the mechanism remains untested and the magnetic component cannot be separated from mechanical or aeration effects. Independent seed lots, additional cultivars, airflow-only controls, time-resolved biochemical sampling, and field evaluation are required before DMFE can be recommended as a seed-conditioning practice.

## Figures and Tables

**Table 1:** Three-way factorial ANOVA of early germination, seed vigour, and final germination percentage in aged KDML 105 rice (*Oryza sativa* L.) seeds subjected to dynamic magnetic-field exposure.

Source of Variation	df	EG			SV			GP		
		*F*	*p*	*η*_p_^2^	*F*	*p*	*η*_p_^2^	*F*	*p*	*η*_p_^2^
Storage duration (*A*)	3	3377.49	<0.001 ***	0.991	116.53	<0.001 ***	0.785	162.59	<0.001 ***	0.836
Flux density (*B*)	3	4.30	0.007 **	0.118	0.41	0.749 ns	0.013	2.04	0.113 ns	0.060
Duration (*T*)	2	3.51	0.034 *	0.068	2.77	0.068 ns	0.055	5.06	0.008 **	0.095
*A* × *B*	9	0.22	0.991 ns	0.020	0.93	0.504 ns	0.080	0.66	0.740 ns	0.058
*A* × *T*	6	1.85	0.098 ns	0.104	2.61	0.022 *	0.140	2.23	0.047 *	0.122
*B* × *T*	6	1.04	0.406 ns	0.061	1.06	0.394 ns	0.062	0.24	0.964 ns	0.015
*A* × *B* × *T*	18	0.65	0.855 ns	0.109	0.64	0.857 ns	0.107	0.54	0.934 ns	0.092
Residual	96	—	—	—	—	—	—	—	—	—

Notes: *A*, storage duration; *B*, magnetic flux density; *T*, exposure duration; EG, early germination; SV, seed vigour; GP, final germination percentage; df, degrees of freedom; *η*_p_^2^, partial eta-squared. Significance: *** *p* < 0.001, ** *p* < 0.01, * *p* < 0.05; ns, not significant.

**Table 2:** Three-way factorial ANOVA of seedling-growth traits in aged KDML 105 rice seeds subjected to dynamic magnetic-field exposure.

Source of Variation	df	SL			RL			SFW			RFW			SDW			RDW		
		*F*	*p*	*η*_p_^2^	*F*	*p*	*η*_p_^2^	*F*	*p*	*η*_p_^2^	*F*	*p*	*η*_p_^2^	*F*	*p*	*η*_p_^2^	*F*	*p*	*η*_p_^2^
Storage duration (*A*)	3	85.77	<0.001 ***	0.728	325.65	<0.001 ***	0.911	105.02	<0.001 ***	0.766	150.42	<0.001 ***	0.825	358.80	<0.001 ***	0.918	233.55	<0.001 ***	0.879
Flux density (*B*)	3	7.46	<0.001 ***	0.189	13.77	<0.001 ***	0.301	28.17	<0.001 ***	0.468	30.59	<0.001 ***	0.489	12.13	<0.001 ***	0.275	27.37	<0.001 ***	0.461
Duration (*T*)	2	10.67	<0.001 ***	0.182	15.43	<0.001 ***	0.243	17.08	<0.001 ***	0.262	7.72	<0.001 ***	0.138	7.97	<0.001 ***	0.142	3.74	0.027 *	0.072
*A* × *B*	9	0.13	0.999 ns	0.012	2.76	0.006 **	0.206	0.71	0.695 ns	0.063	2.67	0.008 **	0.200	0.53	0.852 ns	0.047	0.51	0.866 ns	0.045
*A* × *T*	6	0.13	0.991 ns	0.008	2.39	0.034 *	0.130	1.08	0.381 ns	0.063	0.72	0.637 ns	0.043	1.55	0.171 ns	0.088	0.32	0.924 ns	0.020
*B* × *T*	6	4.57	<0.001 ***	0.222	1.21	0.307 ns	0.070	1.14	0.345 ns	0.067	5.44	<0.001 ***	0.254	2.24	0.046 *	0.123	2.81	0.014 *	0.150
*A* × *B* × *T*	18	0.21	1.000 ns	0.038	1.11	0.357 ns	0.172	0.87	0.611 ns	0.141	0.56	0.919 ns	0.095	0.25	0.999 ns	0.045	0.53	0.939 ns	0.090
Residual	96	—	—	—	—	—	—	—	—	—	—	—	—	—	—	—	—	—	—

Notes: *A*, storage duration; *B*, magnetic flux density; *T*, exposure duration; SL, shoot length; RL, root length; SFW and RFW, shoot and root fresh weight; SDW and RDW, shoot and root dry weight; df, degrees of freedom; *η*_p_^2^, partial eta-squared. Residual mean squares (df 96): SL 32.40; RL 27.27; SFW 2.05; RFW 1.48; SDW 0.0624; RDW 0.0250. Significance: *** *p* < 0.001, ** *p* < 0.01, * *p* < 0.05; ns, not significant. Means and Duncan groupings for the 13 conditions within each storage duration, the untreated control and the twelve treated combinations: Table S1.

**Table 3:** Comparative performance of the Logistic, Gompertz, and Reduced Dual-Phase Gompertz (RDPG) models fitted to water-uptake kinetics of KDML 105 rice seeds stored for 10–13 months.

Storage Duration (Months)	Model	Estimated Coefficients (*p*)	*R* ^2^	RMSE	MAE	RSS	AIC_c_	ΔAIC_c_	AIC	BIC	Rank
10	RDPG	8	0.998	0.54	0.39	28.5	−96.6	0.0	−98.7	−75.7	1
	Gompertz	3	0.936	3.29	2.55	1036.6	236.9	333.5	236.4	246.7	2
	Logistic	3	0.926	3.53	2.67	1196.2	250.6	347.2	250.2	260.4	3
11	RDPG	8	0.997	0.66	0.50	41.8	−59.7	0.0	−61.7	−38.7	1
	Gompertz	3	0.927	3.51	2.63	1179.7	249.3	308.9	248.8	259.1	2
	Logistic	3	0.908	3.95	2.86	1494.7	272.0	331.6	271.6	281.8	3
12	RDPG	8	0.998	0.51	0.38	24.6	−110.6	0.0	−112.7	−89.6	1
	Gompertz	3	0.942	3.12	2.67	937.1	227.2	337.8	226.7	237.0	2
	Logistic	3	0.939	3.23	2.61	1000.7	233.5	344.1	233.0	243.3	3
13	RDPG	8	0.998	0.55	0.39	29.0	−94.7	0.0	−96.8	−73.7	1
	Gompertz	3	0.970	2.27	1.89	493.1	165.5	260.2	165.1	175.4	2
	Logistic	3	0.968	2.36	1.90	534.5	173.3	268.0	172.8	183.1	3

Notes: *p* is the number of estimated mean-model coefficients and K = *p* + 1 includes the residual-variance parameter. ΔAICc is relative to the lowest AICc within each storage duration, and bold marks the first-ranked model; AIC and BIC are secondary criteria. RDPG, Reduced Dual-Phase Gompertz; RMSE, root-mean-square error; MAE, mean absolute error; RSS, residual sum of squares; AICc, small-sample-corrected Akaike information criterion; BIC, Bayesian information criterion.

**Table 4:** Storage-duration-specific coefficient estimates for the selected Reduced Dual-Phase Gompertz (RDPG) model fitted to treated KDML 105 rice seeds.

Coefficient	Role in the Selected RDPG Structure	Estimate (Approximate 95% CI) by Storage Duration (Months)			
		10	11	12	13
*A*_10_	Reference *A*_1_ at *B* = 315 mT and *T* = 6 min (%)	19.518 (19.250, 19.785)	23.838 (23.468, 24.207)	33.084 (32.807, 33.360)	35.809 (35.568, 36.051)
*A*_1*B*_	Change in *A*_1_ per 100 mT (percentage points)	−0.219 (−0.325, −0.113)	−0.041 (−0.169, 0.087)	−0.092 (−0.189, 0.006)	−0.161 (−0.267, −0.054)
*A*_1*T*_	Change in *A*_1_ per minute (percentage points min^−1^)	0.391 (0.304, 0.477)	0.303 (0.189, 0.417)	0.204 (0.119, 0.290)	0.335 (0.253, 0.417)
*K*_1_	Earlier-component rate coefficient (h^−1^)	0.674 (0.614, 0.734)	0.676 (0.613, 0.739)	0.969 (0.891, 1.047)	0.735 (0.697, 0.772)
*A*_20_	Reference *A*_2_ at *T* = 6 min (%)	21.169 (20.653, 21.685)	18.957 (18.146, 19.767)	9.990 (9.378, 10.603)	5.981 (5.616, 6.345)
*A*_2*T*_	Change in *A*_2_ per minute (percentage points min^−1^)	−0.139 (−0.322, 0.044)	−0.406 (−0.679, −0.133)	−0.014 (−0.221, 0.193)	−0.371 (−0.510, −0.232)
*K*_20_	Reference *K*_2_ at *T* = 6 min (h^−1^)	0.201 (0.189, 0.214)	0.161 (0.145, 0.177)	0.164 (0.141, 0.187)	0.479 (0.341, 0.616)
*K*_2*T*_	Change in *K*_2_ per minute (h^−1^ min^−1^)	−0.008 (−0.013, −0.003)	0.002 (−0.004, 0.008)	−0.006 (−0.015, 0.003)	0.089 (0.043, 0.136)

Notes: Values are coefficient estimates with approximate model-based 95% confidence intervals from the storage duration-specific nonlinear least-squares fits. Because *B*_c_ = (*B* − 315)/100 and *T*_c_ = *T* − 6, *A*_1*B*_ is expressed per 100 mT and the duration slopes per minute. *K*_1_ was constant within each fit. The intervals come from the covariance approximation and should be read together with the 95% condition-resampling percentile intervals in Section 2.4, which describe sensitivity to which treatment conditions enter the fit and are not confidence intervals in the full experimental sense. Logistic and Gompertz coefficients: Table S2; bootstrap stability: Table S7. Anchor sensitivity: Figure S5 and Table S6.

**Table 6:** Final reduced response-surface models and adequacy statistics for seedling-growth traits of aged KDML 105 rice seeds under treated-only dynamic magnetic-field exposure.

**(A) Model Structure and Adequacy Statistics**									
**Trait**	**Retained Terms**	**No. of Coefficients**	***R* ^2^**	**Adj. *R*^2^**	**RMSE**	**MAE**	**AIC**	**Model *p***	**Residual Diagnostics**
SL	*A*, *B*, *T*, *B*^2^, *T*^2^	6	0.670	0.658	5.65	4.72	919.4	<0.001	Assumptions satisfactory
RL	*A*, *B*, *T*, *A*^2^, *B*^2^, *T*^2^, *A* × *T*	8	0.843	0.835	6.00	4.70	940.7	<0.001	Assumptions satisfactory
SFW	*A*, *B*, *T*, *B*^2^, *T*^2^, *A* × *T*	7	0.772	0.762	1.35	1.03	509.8	<0.001	Assumptions satisfactory
RFW	*A*, *B*, *T*, *A*^2^, *B*^2^, *B* × *T*	7	0.769	0.759	1.31	1.06	501.1	<0.001	Mild heteroscedasticity (Breusch–Pagan *p* = 0.004)
SDW	*A*, *B*, *T*, *B*^2^, *T*^2^, *A* × *T*, *B* × *T*	8	0.898	0.893	0.24	0.19	8.2	<0.001	Assumptions satisfactory
RDW	*A*, *B*, *T*, *B*^2^, *B* × *T*	6	0.835	0.829	0.16	0.14	−103.1	<0.001	Minor departure from normality (Shapiro–Wilk *p* = 0.027)
**(B) Final Reduced Response-Surface Equations in Natural Units**									
**Trait**	**Final Reduced Response-Surface Equation**								
SL	SL = 153.63 − 6.7789·*A* + 0.080956·*B* + 4.6092·*T* − 0.00011933·*B*^2^ − 0.32631·*T*^2^								
RL	RL = 31.888 + 28.346·*A* + 0.066649·*B* − 3.4367·*T* − 1.8715·*A*^2^ − 0.00013891·*B*^2^ − 0.24767·*T*^2^ + 0.47999·*A*·*T*								
SFW	SFW = 57.987 − 1.3768·*A* + 0.033550·*B* + 2.8391·*T* − 6.5196 × 10^−5^·*B*^2^ − 0.16010·*T*^2^ − 0.085292·*A*·*T*								
RFW	RFW = 69.617 − 7.0682·*A* + 0.053426·*B* + 0.38284·*T* + 0.22396·*A*^2^ − 8.2016 × 10^−5^·*B*^2^ − 0.00076772·*B*·*T*								
SDW	SDW = 11.601 − 0.70567·*A* + 0.0064060·*B* + 0.071336·*T* − 9.6940 × 10^−6^·*B*^2^ − 0.015856·*T*^2^ + 0.016194·*A*·*T* − 0.00015036·*B*·*T*								
RDW	RDW = 5.8910 − 0.30531·*A* + 0.0064459·*B* + 0.054783·*T* − 9.6873 × 10^−6^·*B*^2^ − 0.00013159·*B*·*T*								

Notes: *A*, *B* and *T* are storage duration (months), magnetic flux density (mT) and exposure duration (min); the equations in (B) are in natural units and follow the term order of (A): linear, quadratic, interaction. Coefficients are given to five significant figures, which reproduces the fitted models to within 1.5 × 10^−4^ of the response range. Model *p* is the overall *F*-test. AIC is not comparable between rows because the responses differ in unit and scale. RMSE and MAE are in the unit of the response (mm for SL and RL; mg seedling^−1^ for the four weight responses). SL, shoot length; RL, root length; SFW and RFW, shoot and root fresh weight; SDW and RDW, shoot and root dry weight; *R*^2^, coefficient of determination; RMSE, root-mean square error; MAE, mean absolute error; AIC, Akaike information criterion. Full coefficients, standard errors and term-level *p*-values: Table S4.

## Data Availability

The raw data supporting the conclusions of this article will be made available by the authors on request.

## Supplementary Materials

The following supporting information can be downloaded at https://www.mdpi.com/article/10.3390/plants15203131/s1.

## Abbreviations

The following abbreviations are used in this manuscript:

AIC	Akaike information criterion
AICc	Akaike information criterion corrected for small samples
ANOVA	Analysis of variance
BIC	Bayesian information criterion
DMFE	Dynamic magnetic-field exposure
EG	Early germination
GP	Final germination percentage
KDML 105	Khao Dawk Mali 105
MAE	Mean absolute error
RDPG	Reduced Dual-Phase Gompertz
RDW	Root dry weight
RFW	Root fresh weight
RL	Root length
RMSE	Root-mean-square error
RSM	Response-surface methodology
RSS	Residual sum of squares
SDW	Shoot dry weight
SFW	Shoot fresh weight
SL	Shoot length
SV	Seed vigour
WA	Water uptake
