# Supplementary material for: Storage-Dependent Responses of KDML 105 Rice (Oryza sativa L.) Seeds Across Dynamic Magnetic-Field Treatment Conditions: Germination, Seedling Growth, and Dual-Phase Hydration Modelling
Source: Plants (Basel). 2026 Oct 13;15(20):3131. doi: 10.3390/plants15203131 (PMC13709179; doi:10.3390/plants15203131)
Supplement: Supplementary file 1 [file plants-15-03131-s001.zip › Supplementary_Captions_and_Numbering_Round1.pdf]

# Supplementary Materials — captions and numbering

Storage-Dependent Responses of KDML 105 Rice Seeds across Dynamic Magnetic-Field Treatment Conditions: Germination, Seedling Growth, and Dual-Phase Hydration Modelling. Manuscript plants-4559125, revised version. Figures and tables are numbered independently, each in order of first citation in the revised manuscript. Figures S1–S4 and Tables S1–S4 keep their numbers and are unchanged from the original submission, except that Table S3 now labels its early-germination rows as the percentage-scale comparator and points to Table S9; Figures S5–S6 and Tables S5–S10 are new.

### Statement for insertion in the manuscript

Supplementary Materials. The following are available online: Figure S1, observed against predicted water uptake for the Logistic, Gompertz and Reduced Dual-Phase Gompertz models, shown separately for seed stored 10, 11, 12 and 13 months; Figure S2, residuals against fitted values for the same three water-uptake models and storage durations, on a common vertical scale; Figure S3, observed against predicted values for the final reduced response-surface models of early germination, seed vigor and final germination percentage, fitted to the 144 treated observations; Figure S4, residuals against fitted values for the same three germination models; Figure S5, sensitivity of the Reduced Dual-Phase Gompertz (RDPG) model ranking to the component-location anchors $\tau 1$ and $\tau 2$; Figure S6, predicted early-germination response surfaces over storage duration and magnetic flux density at exposure durations of (a) 3, (b) 6 and (c) 9 min; Table S1, treatment-combination means of the six seedling-growth traits; Table S2, coefficient estimates, with approximate model-based 95% confidence intervals, for the single-component Logistic and Gompertz benchmark models fitted at each storage duration by one-stage nonlinear least squares to the 96 treated condition-by-time means; Table S3, full response-surface coefficients, standard errors, t statistics and term-level p-values for the germination responses, with predictors centred at $A_0$ = 11.5 months, $B_0$ = 315 mT and $T_0$ = 6 min, together with the final equations in natural units; Table S4, the corresponding values for the six seedling-growth traits; Table S5, descriptive comparisons between the untreated control and treated conditions; Table S6, anchor-sensitivity grid for the Reduced Dual-Phase Gompertz model: ΔAICc summarised over the 153 anchor pairs at each storage duration, for the re-selected and the fixed-structure variant, with the seventeen pairs at $\tau 1$ = 6.0 h listed in full; Table S7, bootstrap stability of the flux-density dependence of the earlier-component amplitude (A1B) and the exposure-duration dependence of the later-component rate coefficient (K2T): percentile intervals and sign retention from 10,000 fixed-structure resamples per storage duration, term-retention frequency with Wilson intervals from 1000 re-selections, and leave-one-condition-out influence; Table S8, grouped cross-validated prediction error for the germination responses on the percentage and the arcsine-square-root scale: repeated grouped 8-fold cross-validation over 200 random partitions, for all three responses and for the four early-germination pipelines; Table S9, coefficients, joint tests and model adequacy for the early-germination model on the arcsine-square-root scale, the model reported in Table 5A; Table S10, drop-one-flux-level diagnostic for the quadratic flux-density term of the early-germination model on the arcsine-square-root scale, with the retention of that term under four resampling schemes.

## Figures

**Figure S1.** Observed against predicted water uptake for the Logistic, Gompertz and Reduced Dual-Phase Gompertz models, shown separately for seed stored 10, 11, 12 and 13 months. Points are the 96 treated condition-by-time means at each storage duration; the diagonal is the line of identity. Cited in Section 2.4.

**Figure S2.** Residuals against fitted values for the same three water-uptake models and storage durations, on a common vertical scale. Cited in Section 2.4, in support of the reported departures from residual normality at 10 and 13 months.

**Figure S3.** Observed against predicted values for the final reduced response-surface models of early germination, seed vigor and final germination percentage, fitted to the 144 treated observations. Cited in Section 2.5.

**Figure S4.** Residuals against fitted values for the same three germination models. Cited in Section 2.5, in support of the reported heteroscedasticity near the upper percentage bound for seed vigor and final germination percentage.

**Figure S5.** Sensitivity of the Reduced Dual-Phase Gompertz (RDPG) model ranking to the component-location anchors $\tau 1$ and $\tau 2$. $\Delta$AICc of the RDPG against the better of the Logistic and Gompertz benchmarks, over $\tau 1$ = 2.0–6.0 h × $\tau 2$ = 18.0–26.0 h in 0.5 h steps, shown separately for seed stored 10, 11, 12 and 13 months. Positive values, in blue, favour the RDPG. The RDPG structure is re-selected at each anchor pair; the fixed-structure variant is summarised in Table S6(A). Contours mark $\Delta$AICc = 0 and 10 and the star marks the published anchors, $\tau 1$ = 3 h and $\tau 2$ = 22 h. Cited in Section 2.4.

**Figure S6.** Predicted early-germination response surfaces over storage duration and magnetic flux density at exposure durations of (a) 3, (b) 6 and (c) 9 min. Predictions were obtained on the arcsine-square-root scale from the model of Table 5A and inverse-transformed to percentages. The three panels share one colour scale so that they can be compared directly. Open circles mark the sampled storage-duration × flux-density combinations. Cited in Section 2.5.

## Tables

**Table S1.** Treatment-combination means of the six seedling-growth traits. Values are means ± standard deviation of three independent replicates. Within each storage duration and trait, different lowercase letters indicate significant differences among the thirteen treatment combinations (untreated control and twelve magnetic-field treatments) by Duncan's multiple range test at $\alpha$ = 0.05. Cited in Section 2.3.

**Table S2.** Coefficient estimates, with approximate model-based 95% confidence intervals, for the single-component Logistic and Gompertz benchmark models fitted at each storage duration by one-stage nonlinear least squares to the 96 treated condition-by-time means. Under the common structure selected by minimising the summed AICc, neither benchmark retained a dependency on magnetic flux density or exposure duration, so each has three constant coefficients per storage duration. Cited in Section 2.4.

**Table S3.** Full response-surface coefficients, standard errors, t statistics and term-level p-values for the germination responses, with predictors centred at $A_0$ = 11.5 months, $B_0$ = 315 mT and $T_0$ = 6 min, together with the final equations in natural units. Cited in Section 2.5, as the companion to Table 5.

**Table S4.** The corresponding values for the six seedling-growth traits. Cited in Section 2.6, as the companion to Table 6.

**Table S5.** Descriptive comparisons between the untreated control and treated conditions. Panels (A)–(C) present early germination, seed vigor and final germination percentage for each treated combination within each storage duration, as means ± standard deviation of three independent replicates, with Duncan groupings at $\alpha$ = 0.05. Panel (D) presents the untreated control alongside the treated mean and range at each

imbibition time for water uptake. The control received neither magnetic exposure nor vortex airflow. Cited in Sections 2.1, 2.2 and 4.3.

**Table S6.** Anchor-sensitivity grid for the Reduced Dual-Phase Gompertz model: ΔAICc summarised over the 153 anchor pairs at each storage duration, for the re-selected and the fixed-structure variant, with the seventeen pairs at $\tau 1 = 6.0$ h listed in full. Cited in Section 2.4.

**Table S7.** Bootstrap stability of the flux-density dependence of the earlier-component amplitude (A1B) and the exposure-duration dependence of the later-component rate coefficient (K2T): percentile intervals and sign retention from 10,000 fixed-structure resamples per storage duration, term-retention frequency with Wilson intervals from 1000 re-selections, and leave-one-condition-out influence. Cited in Section 2.4.

**Table S8.** Grouped cross-validated prediction error for the germination responses on the percentage and the arcsine-square-root scale: repeated grouped 8-fold cross-validation over 200 random partitions, for all three responses and for the four early-germination pipelines. RMSE is in percentage points. Cited in Section 2.5.

**Table S9.** Coefficients, joint tests and model adequacy for the early-germination model on the arcsine-square-root scale, the model reported in Table 5A. Cited in Section 2.5, as the companion to Table 5A.

**Table S10.** Drop-one-flux-level diagnostic for the quadratic flux-density term of the early-germination model on the arcsine-square-root scale, with the retention of that term under four resampling schemes. Cited in Section 2.5.

## Numbering record

The original submission contained Figures S1–S4 and Tables S1–S4. The Supplementary Materials statement of the submitted manuscript listed only the four tables and omitted the four figures; that omission is corrected in the revised manuscript, which lists all six figures and all ten tables. Citations of Figures S1–S4, which the submitted manuscript never carried, are added at the hydration residual-diagnostics sentence of Section 2.4 (Figures S1 and S2) and the germination residual-diagnostics sentence of Section 2.5 (Figures S3 and S4). No previously numbered item is renumbered.
