# Supplementary material for: Storage-Dependent Responses of KDML 105 Rice (Oryza sativa L.) Seeds Across Dynamic Magnetic-Field Treatment Conditions: Germination, Seedling Growth, and Dual-Phase Hydration Modelling
Source: Plants (Basel). 2026 Oct 13;15(20):3131. doi: 10.3390/plants15203131 (PMC13709179; doi:10.3390/plants15203131)
Supplement: Supplementary file 1 [file plants-15-03131-s001.zip › Supplementary_Materials_Round1_TablesS5-S10.pdf]

Supplementary Materials

Storage-Dependent Responses of KDML 105 Rice Seeds across Dynamic Magnetic-Field Treatment Conditions: Germination, Seedling Growth, and Dual-Phase Hydration Modelling. Manuscript plants-4559125. This file contains Tables S5–S10. Tables S1–S4 and Figures S1–S6 are supplied as separate files: Figure S5 is the anchor-sensitivity grid for the Reduced Dual-Phase Gompertz model and Figure S6 the early-germination response surfaces at exposure durations of 3, 6 and 9 min. Table S5 summarises the untreated control alongside the corresponding treated descriptive results. Tables S6–S10 report analyses of the 144 treated replicate-level observations, or of the 96 treated condition-by-time means, used in the main analyses.

Table S5. Untreated control against every treated combination, by storage duration. Values are means ± standard deviation of three independent replicates for early germination (EG, day 2), seed vigor (SV, day 5) and final germination percentage (GP, day 14), and mean water uptake for the imbibition series. Treatments are labelled flux density / exposure duration, in mT and min. Within each storage duration and response, values sharing a letter do not differ by Duncan's multiple range test at $\alpha = 0.05$; the error term is that storage duration's one-way residual (df = 26). The untreated control received neither magnetic exposure nor vortex airflow, so a control–treated difference confounds the magnetic component with mechanical, aeration and chamber-passage effects.

(A) Early germination, EG (%)

Treatment	10 M	11 M	12 M	13 M
Control	78.0±3.0 d	51.7±7.4 c	17.0±2.6 b	6.0±1.0 b
150/3	93.0±2.6 abc	74.0±1.0 ab	31.0±5.3 a	12.3±2.5 ab
150/6	96.3±1.5 a	77.7±2.1 ab	34.0±5.3 a	14.3±4.7 a
150/9	95.3±0.6 ab	74.7±2.5 ab	29.3±5.5 a	18.3±2.1 a
270/3	94.7±3.8 ab	76.0±7.9 ab	29.7±3.5 a	16.0±7.0 a
270/6	96.3±0.6 a	81.0±3.6 a	33.3±6.7 a	18.7±1.5 a
270/9	95.3±0.6 ab	74.3±5.5 ab	30.7±6.1 a	14.0±4.6 a
370/3	94.7±0.6 ab	77.7±6.0 ab	34.3±2.5 a	19.0±2.6 a
370/6	96.7±2.5 a	81.3±3.2 a	33.3±4.5 a	14.7±2.5 a
370/9	95.7±1.5 ab	73.7±2.5 ab	31.3±6.8 a	17.0±3.6 a
470/3	92.0±2.0 bc	77.0±2.6 ab	30.0±5.6 a	13.3±4.5 a
470/6	95.3±1.2 ab	77.7±3.5 ab	26.3±1.5 a	12.3±1.5 a
470/9	90.7±1.2 c	72.0±2.0 b	29.7±4.2 a	15.7±4.2 a

(B) Seed vigor, SV (%)

Treatment	10 M	11 M	12 M	13 M
Control	96.7±2.5 a	95.0±2.6 a	89.7±5.5 b	82.7±6.0 b
150/3	97.0±2.6 a	97.7±0.6 a	93.7±0.6 ab	91.3±3.1 a
150/6	99.3±0.6 a	95.3±2.3 a	94.3±2.5 ab	88.0±1.0 ab
150/9	98.7±1.2 a	95.7±2.1 a	90.3±2.5 b	89.0±1.7 a
270/3	97.7±2.1 a	95.3±1.5 a	92.7±1.2 ab	92.3±0.6 a
270/6	98.7±0.6 a	96.7±0.6 a	95.7±1.2 a	90.3±1.5 a
270/9	98.3±1.5 a	96.0±2.0 a	91.0±3.6 ab	89.0±2.6 a
370/3	98.0±1.0 a	97.7±2.1 a	92.3±2.1 ab	91.3±3.2 a
370/6	98.0±1.0 a	96.3±0.6 a	90.3±3.1 b	88.3±5.5 ab
370/9	99.3±1.2 a	96.7±1.5 a	90.3±3.1 ab	90.7±0.6 a
470/3	98.3±0.6 a	96.3±0.6 a	92.0±2.0 ab	91.7±4.0 a
470/6	98.0±1.0 a	97.0±1.7 a	90.7±2.3 ab	89.3±1.2 a
470/9	98.7±0.6 a	97.0±2.0 a	91.0±2.0 ab	87.0±5.3 ab

(C) Final germination percentage, GP (%)

Treatment	10 M	11 M	12 M	13 M
Control	94.3±2.1 ab	90.7±2.1 b	84.0±2.0 a	81.3±5.5 ab
150/3	94.7±1.5 ab	93.3±1.2 ab	88.0±5.3 a	82.7±2.5 ab
150/6	97.0±1.0 a	93.3±0.6 ab	87.7±2.9 a	83.7±4.5 ab

Treatment	10 M	11 M	12 M	13 M
150/9	96.3±0.6 ab	91.3±3.1 ab	84.7±2.1 a	81.7±1.5 ab
270/3	94.3±2.5 ab	92.7±4.0 ab	87.0±4.6 a	82.7±2.5 ab
270/6	96.3±1.5 ab	93.3±2.1 ab	86.7±3.1 a	80.3±3.2 ab
270/9	95.3±1.5 ab	91.3±1.5 ab	84.7±1.2 a	79.0±5.3 b
370/3	94.0±1.0 b	95.7±2.1 a	86.7±4.0 a	86.3±2.1 a
370/6	96.3±0.6 ab	92.0±1.0 ab	89.0±3.0 a	84.7±2.5 ab
370/9	96.3±1.5 ab	92.3±2.1 ab	83.0±2.6 a	82.0±4.6 ab
470/3	94.3±1.5 ab	91.3±3.1 ab	87.7±2.5 a	83.7±4.0 ab
470/6	95.7±0.6 ab	93.3±2.5 ab	84.3±2.5 a	80.3±2.5 ab
470/9	95.3±1.5 ab	93.3±3.1 ab	82.7±7.0 a	79.3±3.1 ab

(D) Water uptake, WA (%), untreated control against the treated mean and range

Storage (months)	Series	0 h	6 h	12 h	18 h	24 h	30 h	36 h	42 h
10	control	0.00	12.21	14.38	18.79	27.66	35.02	38.05	39.40
	treated mean	0.00	17.10	19.60	21.90	29.77	37.44	39.06	40.44
	treated range	0.00–0.00	15.36–18.54	17.86–21.05	19.64–23.90	28.47–31.31	36.31–38.78	37.90–40.11	39.21–41.34
11	control	0.00	18.04	20.56	22.90	29.51	37.10	39.55	40.68
	treated mean	0.00	20.94	23.18	27.45	32.73	39.00	40.92	41.45
	treated range	0.00–0.00	19.60–22.16	21.66–24.56	26.33–28.41	31.70–33.52	38.00–39.77	40.32–41.64	40.86–42.26
12	control	0.00	27.86	29.33	32.07	36.08	40.32	40.96	41.14
	treated mean	0.00	31.33	32.58	35.03	38.09	41.13	41.95	42.34
	treated range	0.00–0.00	30.21–32.01	32.07–33.70	33.72–36.79	36.90–39.57	40.27–41.86	41.28–42.61	41.74–42.81
13	control	0.00	28.02	30.70	32.92	37.81	39.92	40.74	41.18
	treated mean	0.00	32.10	34.95	36.78	39.82	41.30	41.64	41.74
	treated range	0.00–0.00	30.76–33.15	33.86–36.21	35.23–38.64	38.16–41.56	40.29–42.04	40.87–42.59	41.17–42.30

Panels (A)–(C) are reproduced from the frozen Section 3.3 statistical reference for this study. The six seedling-growth traits are not repeated here: Table S1 already reports them for all 52 combinations, that is the untreated control and the twelve treated combinations at each of the four storage durations, with the same within-storage-duration Duncan convention. Panel (D) reports the condition-by-time means used for the hydration modelling; the control series was held out of model estimation because it is not crossed with the treatment factors.

Table S6. Anchor-sensitivity grid for the Reduced Dual-Phase Gompertz (RDPG) model. ΔAICc of the RDPG against the better of the Logistic and Gompertz benchmarks at each storage duration, over τ_1 = 2.0–6.0 h × τ_2 = 18.0–26.0 h in 0.5 h steps (153 anchor pairs; 235,008 candidate fits). Positive values favour the RDPG. Re-selected chooses the RDPG structure afresh at each anchor pair; fixed refits the published structure. The published anchors are τ_1 = 3 h, τ_2 = 22 h.

(A) Summary over the whole grid

Variant	Storage (months)	min ΔAICc	median	max	cells > 10	cells ≤ 0
re-selected	10	2.9	195.8	338.5	146/153	0/153
re-selected	11	4.5	267.0	336.4	141/153	0/153
re-selected	12	−103.0	322.7	373.8	136/153	17/153
re-selected	13	−164.7	250.2	323.9	136/153	17/153
fixed	10	−2.6	193.8	337.4	145/153	6/153
fixed	11	−3.6	262.6	332.5	136/153	10/153
fixed	12	−111.8	320.3	370.1	136/153	17/153
fixed	13	−173.2	250.2	319.6	136/153	17/153

(B) The τ_1 = 6.0 h boundary, re-selected structure

τ_1 / τ_2 (h)	10 months	11 months	12 months	13 months
6.0 / 18.0	2.9	4.5	−95.5	−151.4
6.0 / 18.5	3.0	4.5	−96.9	−153.0
6.0 / 19.0	3.1	4.6	−92.3	−156.0
6.0 / 19.5	3.2	4.6	−95.1	−155.5
6.0 / 20.0	3.3	4.6	−96.1	−156.6
6.0 / 20.5	4.0	4.7	−97.1	−157.5
6.0 / 21.0	8.3	4.7	−98.0	−158.5
6.0 / 21.5	12.6	4.7	−103.0	−161.0
6.0 / 22.0	16.7	5.3	−103.0	−161.9
6.0 / 22.5	20.8	6.7	−103.0	−162.6
6.0 / 23.0	24.5	8.1	−103.0	−163.3
6.0 / 23.5	27.8	9.6	−103.0	−164.0
6.0 / 24.0	30.4	11.0	−103.0	−162.8
6.0 / 24.5	32.2	15.7	−103.0	−163.4
6.0 / 25.0	32.9	15.7	−103.0	−163.9
6.0 / 25.5	32.7	15.7	−103.0	−164.3
6.0 / 26.0	31.7	16.1	−103.0	−164.7

The RDPG leads by more than 10 AICc units at every storage duration in 136 of the 153 anchor pairs, comprising τ_1 = 2.0–5.5 h with τ_2 = 18–26 h, where the smallest margin is 117.2. The 17 pairs that fail all have τ_1 = 6.0 h and are listed in panel (B): there the RDPG remains preferred at 10 and 11 months but not at 12 and 13 months. As τ_1 approaches 6 h the fitted K_1 rises sharply while the residual sum of squares is unchanged, which indicates weak joint identification of the earlier component rather than a better fit: the component takes the value A_1/e at $t = \tau_1$ whatever K_1 is, so placing the anchor on the only post-baseline observation of the rapid phase forces an amplitude above the observed plateau. The full 153-cell grid for the re-selected variant is shown in Figure S5, whose four panels are the four storage durations; the fixed-structure variant is summarised in panel (A) of this table.

Table S7. Bootstrap stability of the two treatment-dependent coefficients of the selected Reduced Dual-Phase Gompertz structure: the flux-density dependence of the earlier-component amplitude (A_1B) and the exposure-duration dependence of the later-component rate coefficient (K_2T). The resampling unit is the treatment condition, drawn with replacement, each contributing its complete eight-point trajectory. Percentile intervals and sign retention are from 10,000 fixed-structure resamples per storage duration; term retention is from a separate run of 1000 resamples per storage duration in which the structure is re-selected from all 256 candidates.

(A) A_1B, per 100 mT

Storage (months)	Point estimate	2.5%	97.5%	Excludes 0	Sign retained	Term retained	95% Wilson interval
10	−0.2189	−0.4148	−0.0103	yes	97.9%	87.7%	85.5–89.6%
11	−0.0411	−0.1654	0.1070	no	70.7%	21.5%	19.1–24.2%
12	−0.0916	−0.2574	0.0826	no	89.8%	43.2%	40.2–46.3%
13	−0.1609	−0.3430	0.0315	no	95.9%	81.1%	78.6–83.4%

(B) K_2T, per minute

Storage (months)	Point estimate	2.5%	97.5%	Excludes 0	Sign retained	Term retained	95% Wilson interval
10	−0.0076	−0.0121	−0.0039	yes	100.0%	97.6%	96.5–98.4%
11	0.0019	−0.0009	0.0046	no	91.9%	4.4%	3.3–5.9%
12	−0.0061	−0.0104	−0.0023	yes	99.6%	75.6%	72.8–78.2%
13	0.0894	0.0035	0.1208	yes	98.8%	97.3%	96.1–98.1%

(C) Leave-one-condition-out influence on A_1B

Storage (months)	Full-data estimate	Range over the 12 refits	Largest single shift
10	−0.2189	−0.3071 to −0.1658	40% (drop 150 mT, 9 min)
11	−0.0411	−0.1073 to 0.0077	161% (drop 150 mT, 9 min)
12	−0.0916	−0.1348 to −0.0471	49% (drop 470 mT, 9 min)
13	−0.1609	−0.2157 to −0.1229	34% (drop 150 mT, 9 min)

Intervals in panels (A) and (B) are 95% condition-resampling percentile intervals. They describe sensitivity to which treatment conditions enter the fit and are not confidence intervals in the full experimental sense, because replicate trajectories were available only for the 10-month group and within-condition variability is therefore not represented. Term retention is from a separate bootstrap of 1000 resamples per storage duration in which the structure is re-selected from all 256 candidates; the Wilson interval is used because the proportions lie near the boundaries. The 10-month A_1B retention is quoted in the text as approximately 88%. At 12 months the K_2T retention depends heavily on one condition, B = 470 mT with T = 9 min: 89.6% in resamples containing it against 49.3% in those without. Of 40,000 fixed-structure resamples none was degenerate, every Jacobian had full rank and no coefficient exceeded 10^3.

Table S8. Grouped cross-validated prediction error for the germination responses on the percentage and arcsine-square-root scales. Repeated grouped 8-fold cross-validation, 200 random partitions; folds are grouped by the 48 A × B × T cells so the three replicates of a condition always fall in the same fold; backward selection is re-run inside every training fold; arcsine predictions are inverse-transformed to percentages before any error is computed; both scales see identical partitions, so every comparison is paired. RMSE is in percentage points.

(A) All three responses, full candidate set

Response	RMSE, percentage	RMSE, arcsine	ΔRMSE (mean), arcsine − percentage	Central 95% range across partitions	Partitions favouring arcsine
EG	8.107 ± 0.225	7.012 ± 0.204	−1.096	−1.372 to −0.732	200/200
SV	2.178 ± 0.018	2.280 ± 0.034	0.102	0.056 to 0.168	0/200
GP	2.895 ± 0.035	2.915 ± 0.032	0.020	−0.016 to 0.057	27/200

(B) Early germination: the four pipelines

Pipeline	RMSE (mean ± SD across partitions)
arcsine, flux-density terms excluded	6.651 ± 0.144
arcsine, flux-density terms allowed	7.012 ± 0.204
percentage, flux-density terms excluded	7.866 ± 0.153
percentage, flux-density terms allowed	8.107 ± 0.225

(C) The cost of allowing flux-density terms

Contrast	ΔRMSE (mean), allowed − excluded	Central 95% range across partitions	Partitions favouring
allowing flux density, arcsine scale	0.361	0.113 to 0.696	0/200
allowing flux density, percentage scale	0.241	0.000 to 0.689	0/200

Sign conventions differ between the panels and are stated in the column headings. In panel (A) ΔRMSE is the arcsine RMSE minus the percentage RMSE, so a negative value favours the arcsine scale. In panel (C) ΔRMSE is the RMSE with flux-density terms allowed minus the RMSE with them excluded, so a positive value means that allowing those terms increased prediction error. The quoted spread is the population standard deviation of the RMSE across the 200 partitions. The partitions share the same 144 observations and overlap heavily, so the spread is descriptive of fold-scheme variability: it is not a standard error and no confidence interval is attached to it. Panel (C) is the test that answers the question directly: inside every outer training fold, backward selection is run twice, once from the full nine-term candidate set and once from a candidate set with every flux-density term removed, so nothing about flux density can reach the second pipeline. Allowing flux-density terms raised prediction error on both scales, in every one of the 200 partitions. An information criterion is not used to choose between the scales because the two models are fitted to different response variables and their likelihoods are not comparable without a Jacobian adjustment.

Table S9. Coefficients of the early-germination model on the arcsine-square-root scale, the model reported in Table 5A. The response is $y^* = \arcsin\sqrt{(EG/100)}$ in radians; predictors are centred at $A_0 = 11.5$ months, $B_0 = 315$ mT and $T_0 = 6$ min. The terms are those the published backward-elimination rule retains on this scale; the rule was not altered.

(A) Coefficients

Term	Estimate	Standard error	t	p
Intercept	0.857614	0.015315	55.998	2.727×10^{-96}
A_c	−0.33021	0.0055665	−59.321	1.383×10^{-99}
B_c	-6.6731×10^{-5}	5.2878×10^{-5}	−1.262	0.2091
T_c	−0.000519785	0.0025408	−0.205	0.8382
A_c^2	0.0222939	0.0062236	3.582	0.0004724
B_c^2	-1.24883×10^{-6}	5.4031×10^{-7}	−2.311	0.02231
T_c^2	−0.00295325	0.0014669	−2.013	0.04605

(B) Joint tests of the retained pairs

Pair	F	df	p
$B_c + B_c^2$	3.15035	(2, 137)	0.04596
$T_c + T_c^2$	2.04751	(2, 137)	0.13299

(C) Model adequacy

Statistic	Transformed scale (radians)	After inverse transformation (percentage points)
R^2	0.9628	—
Adjusted R^2	0.9611	—
RMSE	0.0728	6.00
MAE	0.0599	4.68
AIC	−331.7	—
Model F	590.36 (6, 137)	—
Model p	2.94×10^{-95}	—

The linear B_c and T_c terms were retained to preserve marginality because their quadratic terms were retained; their individual tests in panel (A) represent local slopes at the centring values, and panel (B) gives the joint tests that govern how strongly each effect may be described. AIC is reported only to document model selection and is comparable only between models fitted to the same response on the same scale. The right-hand column of panel (C) is descriptive: it is computed after inverse transformation and is not used as evidence for the choice of scale, which rests on the out-of-sample comparison in Table S8. No coefficient of determination is reported after inverse transformation: a coefficient of determination computed from back-transformed fitted values is not comparable with the least-squares R^2 of the transformed fit, and reporting one invites exactly that comparison, so only RMSE and MAE are given on the percentage scale. All transformed-scale fitted values remained within $[0, \pi/2]$, so inverse transformation required no clipping. In natural units the equation rounds to four significant figures with a largest back-transformed difference of 0.1228 percentage points, which is why Table 5A prints the centred form in which the model was fitted.

Table S10. Drop-one-flux-level diagnostic for the quadratic flux-density term of the early-germination model on the arcsine-square-root scale. Each of the four flux-density levels is removed from the full data in turn and the model re-selected by the same rule.

(A) Terms retained when one flux-density level is removed

Level removed (mT)	Terms retained	B_c^2 retained	B_c^2 coefficient	B_c^2 p
150	{A, A^2}	no	—	—
270	{A, B, A^2, B^2}	yes	-1.73963×10^{-6}	0.0129
370	{A, A^2}	no	—	—
470	{A, T, A^2, T^2}	no	—	—

(B) Retention of B_c^2 under different resampling schemes

Assessment	Training set size	Arcsine	Percentage
Leave-one-cell-out, 48 folds	141	91.7%	2.1%
Repeated grouped 8-fold, 1600 folds	126	61.6%	17.4%
Cell-level bootstrap of the full data, 2000 resamples	144	59.2%	—
Full data, single fit	144	retained, $p = 0.0223$	not retained

Selection of B_c^2 was sensitive to the available flux-density support. No stronger claim is warranted from panel (A): removing a level also removes a quarter of the observations, narrows the range of B, and leaves only three points from which to estimate curvature, so the loss of the term cannot be attributed to the removed level itself. In panel (B) the leave-one-cell-out figure is the outlier because those training sets are nearly identical to one another and to the full data, so they almost all reproduce the full-data selection; both schemes whose training sets overlap less put arcsine retention near 60%. Selection instability affects both scales: with 126 rather than 141 observations the gap between them narrows from about 90 to about 44 percentage points. Separately, on both scales the pipeline that re-runs backward elimination inside every fold predicts worse than the corresponding fixed model (8.107 against 7.680 on the percentage scale, 7.012 against 6.688 on the arcsine scale); the fixed term sets were chosen after seeing all 144 observations and carry their own optimism, so the two levels are not directly comparable.
