# Supplementary material for: Storage-Dependent Responses of KDML 105 Rice (Oryza sativa L.) Seeds Across Dynamic Magnetic-Field Treatment Conditions: Germination, Seedling Growth, and Dual-Phase Hydration Modelling
Source: Plants (Basel). 2026 Oct 13;15(20):3131. doi: 10.3390/plants15203131 (PMC13709179; doi:10.3390/plants15203131)
Supplement: Supplementary file 1 [file plants-15-03131-s001.zip › Table_S1/Table_S1_SeedlingGrowth_TreatmentMeans.pdf]

Supplementary Table S1. *Treatment-combination means of seedling-growth traits of aged KDML 105 rice seeds subjected to dynamic magnetic-field exposure.*

Storage duration (months)	Magnetic flux density (mT)	Exposure duration (min)	n	SL (mm)	RL (mm)	SFW (mg)	RFW (mg)	SDW (mg)	RDW (mg)
**10**	*Control*	0	3	103.81 ± 5.99^{a†}	118.27 ± 2.74^{d}	49.39 ± 0.71^{e}	26.63 ± 0.38^{e}	5.44 ± 0.43^{c}	3.55 ± 0.21^{c}
10	150	3	3	109.44 ± 2.08^{a†}	127.94 ± 5.60^{c}	52.29 ± 0.78^{bcd}	28.22 ± 0.54^{cde}	5.79 ± 0.36^{abc}	3.70 ± 0.11^{bc}
10	150	6	3	112.81 ± 5.12^{a†}	131.71 ± 3.23^{bc}	53.04 ± 2.45^{abc}	28.55 ± 0.20^{cde}	5.74 ± 0.23^{abc}	3.76 ± 0.23^{bc}
10	150	9	3	111.26 ± 4.87^{a†}	126.12 ± 2.77^{c}	54.03 ± 2.65^{ab}	28.34 ± 0.35^{cde}	5.82 ± 0.25^{abc}	3.79 ± 0.05^{bc}
10	270	3	3	109.02 ± 5.41^{a†}	136.98 ± 3.50^{ab}	52.43 ± 0.54^{bcd}	29.30 ± 0.43^{cd}	5.92 ± 0.17^{abc}	3.80 ± 0.09^{bc}
10	270	6	3	112.13 ± 3.13^{a†}	137.59 ± 2.20^{ab}	54.78 ± 1.01^{a}	32.12 ± 3.00^{ab}	6.21 ± 0.15^{a}	4.17 ± 0.13^{a}
10	270	9	3	113.57 ± 4.89^{a†}	128.56 ± 5.01^{c}	52.98 ± 1.10^{abc}	33.01 ± 1.26^{a}	6.13 ± 0.09^{ab}	4.15 ± 0.13^{a}
10	370	3	3	109.55 ± 6.26^{a†}	139.03 ± 2.83^{a}	51.84 ± 0.70^{bcd}	30.38 ± 0.76^{bc}	6.05 ± 0.18^{ab}	3.83 ± 0.05^{bc}
10	370	6	3	120.82 ± 3.05^{a†}	132.89 ± 1.49^{abc}	53.96 ± 0.42^{ab}	33.41 ± 0.21^{a}	6.18 ± 0.26^{a}	3.84 ± 0.22^{bc}
10	370	9	3	117.34 ± 4.15^{a†}	126.59 ± 1.54^{c}	53.12 ± 0.49^{abc}	30.35 ± 1.89^{bc}	5.98 ± 0.28^{ab}	3.93 ± 0.07^{ab}
10	470	3	3	111.40 ± 4.54^{a†}	127.24 ± 2.70^{c}	52.18 ± 0.76^{bcd}	28.16 ± 1.54^{cde}	5.92 ± 0.44^{abc}	3.72 ± 0.20^{bc}
10	470	6	3	112.83 ± 6.13^{a†}	128.89 ± 6.37^{c}	51.47 ± 0.93^{cde}	27.41 ± 1.20^{de}	5.63 ± 0.18^{bc}	3.63 ± 0.30^{bc}
10	470	9	3	109.96 ± 9.17^{a†}	117.09 ± 3.45^{d}	50.50 ± 0.61^{de}	27.75 ± 0.98^{de}	5.61 ± 0.18^{bc}	3.69 ± 0.24^{bc}
**11**	*Control*	0	3	95.70 ± 3.96^{d}	116.60 ± 3.15^{de}	47.29 ± 2.12^{e}	23.25 ± 1.77^{d}	4.84 ± 0.21^{e}	3.39 ± 0.14^{d}
11	150	3	3	100.30 ± 4.40^{bcd}	126.36 ± 2.92^{abcd}	50.82 ± 1.18^{bc}	27.56 ± 0.37^{abc}	5.20 ± 0.32^{cde}	3.45 ± 0.28^{cd}
11	150	6	3	100.49 ± 8.07^{bcd}	129.81 ± 7.30^{abc}	50.91 ± 1.26^{bc}	26.86 ± 2.49^{abc}	5.38 ± 0.24^{abcd}	3.52 ± 0.12^{bcd}
11	150	9	3	104.19 ± 8.27^{abcd}	124.06 ± 3.45^{bcd}	49.75 ± 0.51^{cd}	26.57 ± 1.03^{abc}	5.43 ± 0.33^{abcd}	3.58 ± 0.11^{bcd}
11	270	3	3	96.40 ± 3.70^{cd}	136.45 ± 6.83^{a}	50.78 ± 0.50^{bc}	26.44 ± 0.56^{abc}	5.21 ± 0.08^{bcde}	3.63 ± 0.06^{bcd}
11	270	6	3	101.98 ± 2.20^{bcd}	131.91 ± 7.41^{ab}	53.52 ± 0.72^{a}	28.47 ± 2.08^{ab}	5.73 ± 0.18^{a}	3.74 ± 0.16^{abc}
11	270	9	3	108.50 ± 9.12^{abc}	127.33 ± 7.41^{abcd}	52.55 ± 0.46^{ab}	29.11 ± 1.48^{a}	5.66 ± 0.41^{ab}	3.96 ± 0.26^{a}
11	370	3	3	98.98 ± 2.20^{bcd}	137.33 ± 3.08^{a}	49.91 ± 0.32^{cd}	27.84 ± 2.49^{abc}	5.34 ± 0.09^{abcd}	3.73 ± 0.07^{abc}
11	370	6	3	114.67 ± 9.23^{a}	126.62 ± 2.39^{abcd}	53.05 ± 1.13^{a}	28.52 ± 0.26^{ab}	5.51 ± 0.10^{abc}	3.80 ± 0.10^{ab}
11	370	9	3	110.49 ± 6.31^{ab}	119.11 ± 3.35^{cde}	48.77 ± 2.07^{cde}	28.50 ± 0.17^{ab}	5.38 ± 0.04^{abcd}	3.56 ± 0.15^{bcd}
11	470	3	3	106.28 ± 9.98^{abcd}	121.10 ± 9.44^{bcde}	48.24 ± 0.30^{de}	26.15 ± 0.81^{bc}	5.16 ± 0.28^{cde}	3.59 ± 0.03^{bcd}
11	470	6	3	104.85 ± 3.37^{abcd}	127.36 ± 8.02^{abcd}	49.27 ± 1.49^{cde}	25.56 ± 1.19^{cd}	5.23 ± 0.23^{bcde}	3.49 ± 0.23^{cd}
11	470	9	3	100.90 ± 5.07^{bcd}	112.57 ± 5.27^{e}	48.24 ± 1.04^{de}	25.28 ± 1.33^{cd}	5.01 ± 0.18^{de}	3.45 ± 0.13^{cd}
**12**	*Control*	0	3	86.69 ± 5.92^{c}	100.61 ± 5.06^{a†}	45.90 ± 0.52^{cd}	23.27 ± 0.23^{g}	4.02 ± 0.11^{c}	2.91 ± 0.13^{e}
12	150	3	3	93.41 ± 6.12^{bc}	116.67 ± 6.86^{a†}	49.75 ± 2.05^{ab}	24.24 ± 0.40^{efg}	4.54 ± 0.36^{ab}	2.95 ± 0.04^{cde}
12	150	6	3	98.75 ± 3.53^{ab}	112.71 ± 3.21^{a†}	50.12 ± 2.22^{a}	25.40 ± 0.92^{bcdef}	4.60 ± 0.33^{ab}	3.17 ± 0.11^{abc}
12	150	9	3	96.67 ± 7.90^{abc}	109.19 ± 4.09^{a†}	48.48 ± 2.87^{abcd}	25.69 ± 0.49^{bcde}	4.61 ± 0.18^{ab}	3.16 ± 0.08^{abcd}
12	270	3	3	94.64 ± 5.62^{bc}	109.16 ± 4.87^{a†}	49.57 ± 1.53^{ab}	24.79 ± 0.20^{defg}	4.51 ± 0.17^{ab}	3.17 ± 0.05^{abc}
12	270	6	3	97.90 ± 5.50^{abc}	109.40 ± 6.85^{a†}	50.64 ± 1.75^{a}	26.72 ± 0.67^{abc}	4.94 ± 0.09^{a}	3.34 ± 0.15^{a}
12	270	9	3	100.57 ± 4.89^{ab}	104.10 ± 8.90^{a†}	49.02 ± 0.48^{abc}	27.82 ± 0.54^{a}	4.79 ± 0.10^{ab}	3.35 ± 0.14^{a}
12	370	3	3	94.57 ± 4.11^{bc}	107.74 ± 2.04^{a†}	49.01 ± 0.44^{abc}	25.12 ± 1.54^{cdef}	4.57 ± 0.36^{ab}	3.19 ± 0.07^{abc}
12	370	6	3	107.60 ± 4.94^{a}	105.77 ± 5.16^{a†}	50.90 ± 1.16^{a}	26.94 ± 0.21^{ab}	4.80 ± 0.38^{ab}	3.22 ± 0.25^{ab}
12	370	9	3	102.93 ± 9.47^{ab}	108.32 ± 9.31^{a†}	48.16 ± 2.54^{abcd}	26.44 ± 1.61^{abcd}	4.55 ± 0.25^{ab}	3.17 ± 0.07^{abc}
12	470	3	3	98.31 ± 2.78^{ab}	102.53 ± 2.02^{a†}	46.64 ± 0.72^{bcd}	24.83 ± 1.54^{defg}	4.47 ± 0.12^{abc}	2.92 ± 0.09^{de}
12	470	6	3	95.79 ± 5.97^{bc}	104.48 ± 5.61^{a†}	48.65 ± 2.56^{abcd}	24.64 ± 1.29^{efg}	4.37 ± 0.36^{bc}	3.00 ± 0.14^{bcde}
12	470	9	3	94.50 ± 6.99^{bc}	103.67 ± 4.99^{a†}	45.64 ± 0.81^{d}	23.86 ± 0.39^{fg}	4.39 ± 0.25^{bc}	2.95 ± 0.19^{cde}
**13**	*Control*	0	3	84.08 ± 5.83^{d}	89.07 ± 6.29^{a†}	44.74 ± 2.97^{bcd}	20.29 ± 1.51^{c}	3.72 ± 0.23^{d}	2.73 ± 0.07^{c}

Storage duration (months)	Magnetic flux density (mT)	Exposure duration (min)	n	SL (mm)	RL (mm)	SFW (mg)	RFW (mg)	SDW (mg)	RDW (mg)
13	150	3	3	86.98 ± 3.97^{cd}	102.19 ± 4.49^{a†}	47.91 ± 1.73^{ab}	23.13 ± 1.34^{b}	3.96 ± 0.26^{abcd}	2.91 ± 0.07^{abc}
13	150	6	3	89.25 ± 5.26^{bcd}	98.30 ± 5.05^{a†}	48.46 ± 0.24^{a}	23.58 ± 0.79^{ab}	4.39 ± 0.13^{ab}	2.98 ± 0.15^{abc}
13	150	9	3	92.23 ± 7.09^{bcd}	97.39 ± 3.91^{a†}	46.30 ± 0.59^{abcd}	24.24 ± 0.31^{ab}	4.20 ± 0.35^{abc}	3.02 ± 0.21^{abc}
13	270	3	3	87.13 ± 4.30^{cd}	101.14 ± 4.97^{a†}	47.91 ± 0.93^{ab}	23.60 ± 1.36^{ab}	3.91 ± 0.36^{bcd}	3.00 ± 0.18^{abc}
13	270	6	3	92.83 ± 4.73^{abcd}	101.47 ± 4.01^{a†}	48.19 ± 0.15^{a}	24.62 ± 0.67^{ab}	4.37 ± 0.08^{ab}	3.13 ± 0.09^{a}
13	270	9	3	94.72 ± 4.73^{abc}	96.46 ± 4.45^{a†}	47.80 ± 1.42^{ab}	25.62 ± 2.01^{a}	4.41 ± 0.15^{a}	3.17 ± 0.14^{a}
13	370	3	3	89.33 ± 3.21^{bcd}	91.23 ± 1.99^{a†}	47.69 ± 1.74^{ab}	23.32 ± 1.96^{b}	3.91 ± 0.18^{bcd}	3.00 ± 0.22^{abc}
13	370	6	3	101.63 ± 4.49^{a}	93.61 ± 5.80^{a†}	48.23 ± 0.16^{a}	24.34 ± 0.34^{ab}	4.30 ± 0.11^{ab}	3.09 ± 0.16^{a}
13	370	9	3	97.10 ± 7.79^{ab}	95.63 ± 6.00^{a†}	46.63 ± 2.35^{abc}	24.46 ± 0.68^{ab}	4.27 ± 0.29^{abc}	3.06 ± 0.26^{ab}
13	470	3	3	90.94 ± 2.52^{bcd}	93.59 ± 1.43^{a†}	44.53 ± 0.16^{cd}	23.71 ± 0.49^{ab}	3.82 ± 0.05^{cd}	2.88 ± 0.13^{abc}
13	470	6	3	92.63 ± 2.25^{abcd}	97.14 ± 5.71^{a†}	46.36 ± 1.07^{abcd}	23.19 ± 0.98^{b}	4.16 ± 0.39^{abcd}	2.75 ± 0.24^{bc}
13	470	9	3	88.33 ± 5.31^{bcd}	95.14 ± 8.68^{a†}	43.47 ± 3.37^{d}	23.29 ± 1.06^{b}	3.97 ± 0.33^{abcd}	2.77 ± 0.08^{bc}

Values are means ± SD of three independent treatment replicates (n = 3). Within each storage duration and each trait, different lowercase superscript letters indicate significant differences among the 13 treatment combinations (control + 12 magnetic-field treatments) according to Duncan's multiple range test at $p < 0.05$; treatments sharing at least one letter are not significantly different. The untreated control (0 mT, 0 min) was included as a within-age reference.

SL = shoot length; RL = root length; SFW = shoot fresh weight; RFW = root fresh weight; SDW = shoot dry weight; RDW = root dry weight.

† For storage duration × trait combinations where the within-storage-duration one-way ANOVA across all 13 treatments was not significant ($p \geq 0.05$: storage duration 10 months × SL, p = 0.096; 12 months × RL, p = 0.111; 13 months × RL, p = 0.114), all treatments are shown with the same letter (a†) by convention, since Duncan's post-hoc test is applied only after a significant omnibus F-test.
