# Supplementary material for: Storage-Dependent Responses of KDML 105 Rice (Oryza sativa L.) Seeds Across Dynamic Magnetic-Field Treatment Conditions: Germination, Seedling Growth, and Dual-Phase Hydration Modelling
Source: Plants (Basel). 2026 Oct 13;15(20):3131. doi: 10.3390/plants15203131 (PMC13709179; doi:10.3390/plants15203131)
Supplement: Supplementary file 1 [file plants-15-03131-s001.zip › Table_S2_Logistic_Gompertz_Coefficients.pdf]

Supplementary Table S2. Coefficient estimates for the single-component Logistic and Gompertz benchmark models fitted to treated KDML 105 rice seeds at each storage duration.

Model	Coefficient	Role in the fitted model	10 months	11 months	12 months	13 months
Logistic Eq. (2) $p = 3$	A	Asymptotic water uptake (%)	42.570 (39.932, 45.208)	41.436 (39.443, 43.430)	38.528 (37.759, 39.296)	39.375 (38.813, 39.936)
	k	Rate coefficient (h^{-1})	0.113 (0.094, 0.131)	0.133 (0.110, 0.157)	1.040 (0.060, 2.019)	1.103 (0.104, 2.102)
	t_0	Inflection time (h)	15.083 (13.351, 16.815)	11.063 (9.703, 12.423)	4.591 (3.245, 5.938)	4.656 (3.429, 5.883)
Gompertz Eq. (3) $p = 3$	A	Asymptotic water uptake (%)	44.940 (41.438, 48.442)	42.140 (40.056, 44.224)	38.779 (38.013, 39.545)	39.551 (38.998, 40.103)
	k	Rate coefficient (h^{-1})	0.074 (0.060, 0.087)	0.099 (0.082, 0.115)	0.448 (0.358, 0.539)	0.476 (0.396, 0.555)
	t_i	Inflection time, lag (h)	10.371 (8.859, 11.883)	6.837 (5.838, 7.836)	2.873 (2.250, 3.497)	2.892 (2.378, 3.407)

Notes. Values are coefficient estimates with approximate model-based 95% confidence intervals from the age-specific one-stage nonlinear least-squares fits to 96 treated condition-by-time means; untreated controls were excluded from estimation.

Under the common structure selected by minimising the summed AICc, neither benchmark retained any dependency on magnetic flux density (B) or exposure duration (T); each model therefore has three constant coefficients per storage duration ($p = 3$). Every coefficient of both models is distinguishable from zero, so no significance markers are shown. The corresponding coefficients of the selected Reduced Dual-Phase Gompertz model, which does retain treatment dependencies, are reported in Table 5.

Model forms are Eq. (2) for the Logistic and Eq. (3) for the Gompertz in Section 2.6. Both forms approach zero only asymptotically and therefore predict small positive water uptake at $t = 0$; these intercepts are a property of the functional form rather than an estimate of seed water content. Predictions are valid only for $B = 150$–470 mT, $T = 3$–9 min and $t = 0$–42 h.
